# An Extended ADOP for Performance Evaluation of Single-Frequency Single-Epoch Positioning by BDS/GPS in Asia-Pacific Region

**DOI:** 10.3390/s17102254

**Published:** 2017-09-30

**Authors:** Xin Liu, Shubi Zhang, Qiuzhao Zhang, Wei Yang

**Affiliations:** School of Environment Science and Spatial Informatics, China University of Mining and Technology, No. 1 Daxue Road, Xuzhou 221116, China; cumt2015lx@163.com (X.L.); zhangsbi@vip.sina.com (S.Z.); cumt_yw@hotmail.com (W.Y.)

**Keywords:** GPS, BDS, SFSE, SMRW, ADOP, E-ADOP, ambiguity resolution, short baseline

## Abstract

Single-Frequency Single-Epoch (SFSE) high-precision positioning has always been the hot spot of Global Navigation Satellite System (GNSS), and ambiguity dilution of precision (ADOP) is a well-known scalar measure for success rate of ambiguity resolution. Traditional ADOP expression is complicated, thus the SFSE extended ADOP (E-ADOP), with the newly defined Summation-Multiplication Ratio of Weight (SMRW) and two theorems for short baseline, was developed. This simplifies the ADOP expression; gives a clearer insight into the influences of SMRW and number of satellites on E-ADOP; and makes theoretical analysis of E-ADOP more convenient than that of ADOP, and through that the E-ADOP value can be predicted more accurately than through the ADOP expression for ADOP value. E-ADOP reveals that number of satellites and SMRW or high-elevation satellite are important for ADOP and, through E-ADOP, we studied which factor is dominant to control ADOP in different conditions and make ADOP different between BeiDou Navigation Satellite System (BDS), Global Positioning System (GPS), and BDS/GPS. Based on experimental results of SFSE positioning with different baselines, some conclusions are made: (1) ADOP decreases when new satellites are added mainly because the number of satellites becomes larger; (2) when the number of satellites is constant, ADOP is mainly affected by SMRW; (3) in contrast to systems where the satellites with low-elevation are the majority or where low- and high-elevation satellites are equally distributed, in systems where the high-elevation satellites are the majority, the SMRW mainly makes ADOP smaller, even if there are fewer satellites than in the two previous cases, and the difference in numbers of satellites can be expanded as the proportion of high-elevation satellites becomes larger; and (4) ADOP of BDS is smaller than ADOP of GPS mainly because of its SMRW.

## 1. Introduction

The Single-Frequency Single-Epoch (SFSE) positioning has always been a hot topic because cycle slip does not need to be detected and repaired for single epoch (SE) and receiver structure of single frequency (SF) is simple, which makes it cheap. However, in the last decade, the success rate of fixing ambiguity for SFSE short baseline of a single system was not high [1,2] and, in [2], the success rates of BeiDou Navigation Satellite System (BDS) and Global Positioning System (GPS) are lower than 97.50% and 85.00%, respectively. The BDS regional satellite navigation system officially started to operate and provide services to the Asia-Pacific region on 27 December 2012 [3]. In addition, the global BDS constellation, which is currently in development stage, will consist of five geosynchronous orbit (GEO), three inclined geosynchronous earth orbit (IGSO) and 27 medium earth orbit (MEO) satellites, and it is expected that it will be operational by 2020 [4]. The GPS constellation, which includes only MEO satellites, is different from BDS, which will make the SFSE success rate of fixing ambiguity of GPS different from that of BDS. At the same time, due to the improvement of GPS and the continuous development of BDS, the SFSE positioning performance will also be improved. Recently, many studies have been conducted on SF and SE positioning. The performance of combined BDS, Galileo, QZSS and GPS SF RTK from the perspectives of ambiguity dilution of precision (ADOP), success rate of fixing ambiguities and positioning accuracy for fixed and float inter-system biases (ISB) showed that combined system could improve the success rate of fixing ambiguities and that much higher elevation cut-off angles could be used in combined systems than in custom systems, and ISB-fixed four-system model achieves significantly higher success rate for cut-off angles of 35–40°, compared to the ISB-float counterpart or BDS/GPS [2]. In [2], it was shown that the success rate of fixing ambiguities of BDS for SFSE RTK is higher than that of GPS. The ambiguity resolution performance of SF Real-time kinematic (RTK) model for different GNSS configurations and positioning scenarios was analyzed by the closed form expression of SF ADOP, and simulation results showed a dual-constellation GNSS can dramatically and instantaneously enhance the ambiguity resolution performance of SF RTK; for instance, the success rate can be above 0.99 for 15 km baselines [5]. The performance of SF and Dual Frequency (DF) BDS/GPS SE kinematic positioning was analyzed in [6], and the results showed that, comparing with SF GPS, the availability and reliability of SF BDS/GPS SE kinematic positioning were significantly improved, and its performance was comparable to that of DF GPS. However, the positioning accuracy and success rate of fixing ambiguities of standalone BDS kinematic positioning were a little worse than those of GPS [6]. The analysis of Single Epoch Dual Frequency (SEDF) of BDS and GPS for baseline distance from 5 to 13 km showed that compared to the standalone GPS or BDS, the combined GNSS system can improve the success rate of fixing ambiguities and the positioning accuracy for SE short baselines: furthermore, SE ambiguity resolution performance of GPS is more reliable than that of the standalone BDS [7]. The combined GPS/BDS instantaneous RTK positioning showed that increased strength of combined model make the ambiguity resolution performance and positioning robustness better than standalone BDS or GPS, and the success rate of GPS was higher than that of BDS for SFSE single-baseline RTK in Australia [8,9]. The instantaneous BDS/GPS RTK positioning with high cut-off elevation angles showed that in the combined system much higher cut-off elevations could be used than in custom standalone systems and SF combined system has an ambiguity resolution performance that is similar to that of a dual-frequency single system. In the case of SFSE RTK, the success rate of BDS is higher than that of GPS [10]. The analysis of SF, dual-GNSS versus dual-frequency, single-GNSS with low-cost and high-grade receivers GPS/BDS RTK showed that SF, dual-system with low-cost receivers could give similar or better instantaneous ambiguity resolution and positioning performance than a dual-frequency, single-system with high-grade receivers, particularly for higher cut-off angles [11]. In addition, the BDS success rate for SFSE RTK was higher than that of GPS in the case that the BDS satellite can be accepted normally [11].

In [6,7,8,9], we can find the success rate of fixing ambiguities of BDS is not greater than that of GPS and the success rate of a combined system is higher than that of a single system. However, in [2,10,11], the situation between BDS and GPS is just the opposite: the success rate of fixing ambiguities of BDS is higher than that of GPS system. At the same time, in [2,5,10,11], the closed form of ADOP was used as a scalar measure for success rate of fixing ambiguities. Based on the ADOP and above references, mainly the total number of visible satellites of double system is much larger than that of a single system and the total number of visible BDS satellites is larger than that of GPS that makes success rate of double system and BDS larger than success rate of single system and GPS, respectively. However, this is only one of the reasons that cause the above-mentioned results. Traditional ADOP expression is complicated; thus, based on the ADOP theory and theoretical deduction and experimental analyses, the SFSE Extended ADOP (E-ADOP), with the newly defined Summation-Multiplication Ratio of Weight (SMRW) and two theorems for short baseline, was developed. This simplifies the ADOP expression; gives a clearer insight into the influences of SMRW and number of satellites on E-ADOP; and makes theoretical analysis of E-ADOP more convenient than that of ADOP, and through that the E-ADOP value can be predicted more accurately than through the ADOP expression for ADOP value. E-ADOP reveals the number of satellites and SMRW or number of high-elevation satellites as important factors to ADOP. The high-elevation satellite has the following advantages. The observation of high-elevation satellite is less affected by multipath and ionosphere than observation of low-elevation satellite, which makes high-elevation satellite observation accuracy higher than that of low-elevation satellite, therefore a large proportion of high-elevation satellites can improve system reliability. Based on E-ADOP, we determined the dominant factor between number of satellites and SMRW to control ADOP in different conditions, and we also studied the reason why ADOP of BDS/GPS and BDS is smaller than ADOP of a single system and GPS, respectively. Based on the E-ADOP, ADOP and Position Dilution of Precision (PDOP), the performance of SFSE positioning was evaluated by experiments with different short baselines in Asia-Pacific Region and obtained results showed that E-ADOP has obvious advantages and it is simple and feasible, and the conclusions of empirical analyses are consistent with the conclusions of theoretical analyses which is based on E-ADOP.

The paper is organized as follows. In Section 2, the SFSE mathematical model, which includes the function and stochastic model for both single-and dual-system, is described. In Section 3, the SFSE ADOP theory for short baseline is introduced, and through ADOP and theoretical deduction, E-ADOP for short baseline was developed and two theorems for SFSE short baseline are established. Then, based on the E-ADOP, the factors that mainly affect E-ADOP and ADOP are studied and some important conclusions are made. In Section 4, the SFSE positioning performance is evaluated by experiments with different baselines in both clear sky conditions and complex sky conditions for BDS, GPS and BDS/GPS. Summary and conclusions are given in Section 5.

## 2. SFSE Mathematical Model

### 2.1. Function Model

When the GPS/BDS dual system is used, the unification of coordinate system and time system is needed. From the aspect of coordinate system, for short baseline, the difference of positioning of the same point obtained by World Geodetic System 1984 (WGS84) coordinate system of GPS and Geodetic Coordinate System (CGCS2000) coordinate system of BDS, which is caused by difference in the flattening of reference ellipsoid between two coordinate systems, can be neglected. Therefore, in this paper, the coordinate system is based on WGS84 coordinate system. From the aspect of time system, the time system is unified to GPS Time (GPST). The relationship between GPST and BDS Time (BDT) is defined as GPST = BDT + (1356 week, 14 s) [7,12].

Because the double difference virtual observations can eliminate or greatly reduce most influences of errors, this paper adopts double difference observations: a satellite with the largest elevation angle in the view field is used as a reference satellite, and the double difference observation equations are composed through respectively making subtraction between a single difference observation equation of the reference satellite and single difference observation equations of the rest of the satellites [13].

For the GPS/BDS system, the reference satellites for double difference observation equations are chosen from both GPS and BDS, and the double difference observation equations are formed for each system, and then the double difference observation equations of the dual system are formed by the superposition of double difference observation equations of these two systems. The pseudo-range observation equations of GPS and BDS, and the carrier phase observation equations of GPS and BDS after linearization are shown as Equations (1) and (2), respectively.

(1) $$\begin{array}{c} c^{g}-\rho^{g}-\varepsilon_{r}^{g}=\left[\begin{array}{ccc} l^{g} & m^{g} & n^{g} \end{array}\right]\cdot {\left[\begin{array}{ccc} \delta x & \delta y & \delta z \end{array}\right]}^{T}\\ c^{b}-\rho^{b}-\varepsilon_{r}^{b}=\left[\begin{array}{ccc} l^{b} & m^{b} & n^{b} \end{array}\right]\cdot {\left[\begin{array}{ccc} \delta x & \delta y & \delta z \end{array}\right]}^{T} \end{array}$$

(2) $$\begin{array}{c} \lambda^{g}\varphi^{g}-\rho^{g}-\varepsilon_{h}^{g}=\left[\begin{array}{ccc} l^{g} & m^{g} & n^{g} \end{array}\right]\cdot {\left[\begin{array}{ccc} \delta x & \delta y & \delta z \end{array}\right]}^{T}-\lambda^{g}N^{g}\\ \lambda^{b}\varphi^{b}-\rho^{b}-\varepsilon_{h}^{b}=\left[\begin{array}{ccc} l^{b} & m^{b} & n^{b} \end{array}\right]\cdot {\left[\begin{array}{ccc} \delta x & \delta y & \delta z \end{array}\right]}^{T}-\lambda^{b}N^{b} \end{array}$$

where s in ***c*^s^**, ***φ*^s^**, ***ρ*^s^**, ***l*****^s^**, ***m*^s^**, ***n*^s^**, ***N*^s^**, ***ε***_r_^s^, and ***ε***_h_^s^ represents g (GPS) or b (BDS); ***c*** is the double difference pseudo-range observation vector; φ is the double difference carrier phase observation vector; ***ρ*** is the double difference geometric distance vector; *δx*, *δy*, and *δz* are the corrections of the baseline vector in *x*, *y*, and *z* directions, respectively; ***l***, ***m***, and ***n*** are the double difference cosine vectors from the station to the satellite in *x*, *y*, and *z* directions, respectively; ***N*** is the double difference carrier integer ambiguity vector; *λ* is the carrier phase wavelength; and ***ε***_r_ and ***ε***_h_ are the observed noise vectors for double difference observations of pseudo-range and carrier phase, respectively.

Equations (1) and (2) can be rewritten in the form of matrix:

(3) $$v=\left[\begin{array}{ccc} A^{g} & 0 & 0\\ A^{b} & 0 & 0\\ A^{g} & -\lambda^{g}\cdot I & 0\\ A^{b} & 0 & -\lambda^{b}\cdot I \end{array}\right]\left[\begin{array}{c} da\\ N^{g}\\ N^{b} \end{array}\right]$$

where $v={\left[\begin{array}{cc} \begin{array}{cc} v_{r}^{g} & v_{r}^{b} \end{array} & \begin{array}{cc} v_{h}^{g} & v_{h}^{b} \end{array} \end{array}\right]}^{T}$; $A^{s}=\left[\begin{array}{ccc} l^{s} & m^{s} & n^{s} \end{array}\right]$; $da={\left[\begin{array}{ccc} \delta x & \delta y & \delta z \end{array}\right]}^{T}$; I is the unit matrix; $v_{r}^{s}$ and $v_{h}^{s}$ are the vectors of correction of double difference observations of pseudo-range and carrier phase, respectively; superscript s is equal either to g or b; and subscripts r and h represent pseudo-range and carrier phase, respectively.

### 2.2. Stochastic Model

In this paper, the classic elevation-dependent weighting model is adopted, which means that the larger the elevation angle is, the greater the weight is. The rules of weighting are as follows: (1) carrier phase observations are not correlated with pseudo-range observations, and different satellite observations are independent of each other [14]; (2) since the carrier phase accuracy is several orders of magnitude higher than the pseudo-range accuracy, the relationship between the carrier phase observation weight and the pseudo-range observation weight is $P_{h}=10^{4}P_{r}$, where *P_h_* and *P_r_* are the weights of carrier phase observation and pseudo-range observation, respectively [13]; and (3) the observations of GPS and BDS in these two systems are independent of each other [7,15].

The variance of each non-difference observation is defined by:

(4) q2=1(sinθ)2

where *q*^2^ is the variance of non-difference observation, and θ is the elevation angle.

The covariance matrix of the double difference observations of a single system can be expressed as:

(5) $$\bar{Q}=\left[\begin{array}{cc} \begin{array}{c} \begin{array}{cc} q_{dd1}^{2} & R^{2}\\ R^{2} & q_{dd2}^{2} \end{array}\\ \begin{array}{cc} \vdots & \vdots \\ R^{2}\; & R^{2} \end{array} \end{array} & \begin{array}{c} \begin{array}{cc} \ldots & R^{2}\\ \ldots & R^{2} \end{array}\\ \begin{array}{cc} \ddots & \vdots \\ \ldots & q_{ddn}^{2} \end{array} \end{array} \end{array}\right]$$

(6) $$\begin{array}{c} q_{ddi}^{2}={\left(q_{f}^{re}\right)}^{2}+{\left(q_{u}^{re}\right)}^{2}+{\left(q_{f}^{i}\right)}^{2}+{\left(q_{u}^{i}\right)}^{2}\\ R^{2}={\left(q_{f}^{re}\right)}^{2}+{\left(q_{u}^{re}\right)}^{2} \end{array}$$

where $q_{ddi}^{2}$ is the covariance of the *i*th double difference observation, *R*^2^ is the covariance of the single difference observation between station and rover for the reference satellite, ${\left(q_{f}^{re}\right)}^{2}$ is the variance of non-difference observations for reference satellite in the station, ${\left(q_{u}^{re}\right)}^{2}$ is the variance of non-difference observations for reference satellite in the rover, ${\left(q_{f}^{i}\right)}^{2}$ is the variance of non-difference observations for the *i*th satellite in the station, ${\left(q_{u}^{i}\right)}^{2}$ is the variance of non-difference observations for the *i*th satellite in the rover, and *n* is the number of double difference ambiguity.

Based on the above conditions, the two-system double difference stochastic model is abbreviated as:

(7) $$Q=\left[\begin{array}{cccc} \tau_{r}^{g}{\bar{Q}}^{g} & 0 & 0 & 0\\ 0 & \tau_{r}^{b}{\bar{Q}}^{b} & 0 & 0\\ 0 & 0 & \tau_{h}^{g}{\bar{Q}}^{g} & 0\\ 0 & 0 & 0 & \tau_{h}^{b}{\bar{Q}}^{b} \end{array}\right]$$

where $\tau_{r}$ is the square of code standard deviation and $\tau_{h}$ is the square of phase standard deviation, whose values will be given in the next analysis, and s in $\tau^{s}$ and ${\bar{Q}}^{s}$ represents either g (GPS) or b. (BDS). The least squares method can be applied to Equations (3) and (7) to obtain the floating ambiguity solution and its variance-covariance matrix $Q_{\hat{a}\hat{a}}$, and then the fixed ambiguity solution can be obtained by Lambda [16,17,18].

## 3. SFSE ADOP for Short Baseline

### 3.1. SFSE ADOP in Closed Form for Short Baseline

The ADOP was firstly introduced in [19,20,21] as an easy-to-compute diagnostic scalar used to measure the intrinsic model strength for successful ambiguity resolution.

The ADOP is defined by:

(8) ADOP=|Qa^a^|12n

where $Q_{\hat{a}\hat{a}}$ is the *n*-dimensional ambiguity variance-covariance matrix and |⋅|. denotes the determinant. The unit of ADOP is cycle.

For a single baseline, which is a geometry-based model, the ADOP of stationary- or moving-receiver short-time can be approximated by [20]:

(9) $$\mathrm{ADOP}=\frac{\sqrt{2}{\left|C_{h}\right|}^{\frac{1}{2j}}}{\overset{ˇ}{\lambda }}{\left[\frac{1}{e_{k}^{T}R_{k}^{-1}e_{k}}\right]}^{\frac{1}{2}}{\left[\frac{\sum_{s=1}^{m}w_{s}}{\prod_{s=1}^{m}w_{s}}\right]}^{\frac{1}{2\left(m-1\right)}}{\left[1+\frac{1}{\eta }\right]}^{\frac{1}{2j}}{\left[1+\frac{1}{\delta }\right]}^{\frac{t}{2j\left(m-1\right)}}$$

where *j* is the number of frequencies, λ˘=∏i=1jλi1j; λ is the wavelength, $C_{h}$ is the cofactor matrix of the phase, *k* is the number of epochs; $e_{k}=\left[1,\;\ldots ,\;1\right]$; ***R****_k_* is the temporal correlation matrix; w is the satellite-dependent weight and $w={\left(\sin\theta \right)}^{2}$, which is the reciprocal of $q^{2}$ that has been defined in Equation (4); θ is elevation angle of satellite, *m* is the number of satellites; and scalar η is the function of ratio of the ambiguity-float and -fixed ionospheric variance factors that are conditioned on the ranges. When ionosphere parameter is fixed, η=∞; when ionosphere parameter is floating, η=[μTCr−1μ][μTCh−1μ], where μ is the coefficient matrix of ionospheric parameters, $C_{r}$ is the cofactor matrix of the pseudo-range, *t* is the number of baseline components, δ is the function of priori precision of un-differenced phase, code and ionosphere observations; and when ionosphere parameter is fixed, δ=[ejTCr−1ej][ejTCh−1ej], and ***e****_j_* is similar to ***e****_k_*.

In the SFSE mathematical model, the parameters of Equation (9) are as follows: $\overset{˘}{\lambda }=\lambda_{i}$, *i* = 1 or 2, *k* = 1, *j* = 1, namely $e_{k}=1$ and $e_{j}=1$, and *R_k_* = 1. At the same time, supposing that the double-difference observation model can eliminate ionospheric and tropospheric delay error for short baseline less than 15 km, namely the ionosphere parameter is fixed, then, η=∞, δ=[Cr−1][Ch−1] and *t* is equal to 3 to eliminate the tropospheric delay error.

Consequently, Equation (9) can be rewritten by:

(10) $$\mathrm{ADOP}=\frac{\sqrt{2}{\left|C_{h}\right|}^{\frac{1}{2}}}{\lambda_{i}}{\left[\frac{\sum_{s=1}^{m}w_{s}}{\prod_{s=1}^{m}w_{s}}\right]}^{\frac{1}{2\left(m-1\right)}}{\left[1+\frac{C_{r}}{C_{h}}\right]}^{\frac{3}{2\left(m-1\right)}}$$

Since $\frac{C_{r}}{C_{h}}\approx 10^{4}\gg 1$, the ${\left[1+\frac{C_{r}}{C_{h}}\right]}^{\frac{3}{2\left(m-1\right)}}\approx {\left[\frac{C_{r}}{C_{h}}\right]}^{\frac{3}{2\left(m-1\right)}}$, let $f_{1}=\frac{\sqrt{2}{\left|C_{h}\right|}^{\frac{1}{2}}}{\lambda_{i}}$, $f_{2}={\left[\frac{\sum_{s=1}^{m}w_{s}}{\prod_{s=1}^{m}w_{s}}\right]}^{\frac{1}{2\left(m-1\right)}}$ and $f_{3}={\left[\frac{C_{r}}{C_{h}}\right]}^{\frac{3}{2\left(m-1\right)}}$. Then, Equation (10) can be abbreviated by:

(11) $$\mathrm{ADOP}=f_{1}\cdot f_{2}\cdot f_{3}$$

### 3.2. Success Rate

As it is well known, ADOP provides good approximation of the integer least squares (ILS) ambiguity success rate, therefore, the approximation of ILS success rate is [20]:

(12) $$P_{s,\mathrm{ILS}}\approx P_{s,ADOP}={\left(2\Phi \left(\frac{1}{\mathrm{ADOP}}\right)-1\right)}^{n}$$

where *n* is the number of double-difference ambiguities and it is equal to (*m* − 1). According to Equation (12), ADOP is inversely proportional to success rate.

In general, the integer bootstrapping (IB) success rate is used to evaluate the lower bound of ILS success rate since ILS pull-in region has usually complicated geometry and its success rate is difficult to calculate [18,22]. The lower bound of ILS success rate can be obtained by [18,23]:

(13) $$P_{s,\mathrm{ILS}}\geq P_{s,\mathrm{IB}}=\prod_{i}^{n}\left(2\Phi \left(\frac{1}{2\sigma_{{\hat{a}}_{i/I}}}\right)-1\right)$$

where $\sigma_{{\hat{a}}_{i/I}}$ denotes the conditional standard deviation.

### 3.3. Extended SFSE ADOP for Short Baseline

#### 3.3.1. Theoretical Deduction of Extended SFSE ADOP for Short Baseline

According to Equation (10), the traditional ADOP expression is complicated, and at the same time $f_{2}$ is a power exponential function of m and $\frac{\sum_{s=1}^{m}w_{s}}{\prod_{s=1}^{m}w_{s}}$. To simplify Equation (10), we take the logarithmic function based on *e* for Equation (10), and combine it with Equation (11):

(14) $$\ln\frac{\mathrm{ADOP}}{f_{1}}=\frac{1}{m-1}\left(\ln{\left(\frac{\sum_{s=1}^{m}w_{s}}{\prod_{s=1}^{m}w_{s}}\right)}^{\frac{1}{2}}+\ln{\left(\frac{C_{r}}{C_{h}}\right)}^{\frac{3}{2}}\right)$$

where $0<w_{s}\leq 1$ based on Equation (4).

As is well known, high-elevation satellites have their own advantages. Namely, the observation of high-elevation satellite is less affected by multipath and ionosphere than that of low-elevation satellite which makes its observation accuracy higher than accuracy of low-elevation satellite. Hence, a large proportion of high-elevation satellites can improve system reliability which is important to improve ambiguity success rate. In Equation (14), only factor $\frac{\sum_{s=1}^{m}w_{s}}{\prod_{s=1}^{m}w_{s}}$ is related to satellite elevation which means it is an important factor for ADOP. Thus, $\frac{\sum_{s=1}^{m}w_{s}}{\prod_{s=1}^{m}w_{s}}$ is defined as the Summation-Multiplication Ratio of Weight (SMRW).

The SMRW has two important properties based on the condition that $0<w_{s}\leq 1$.

**Theorem** **1.** *The SMRW becomes larger when new satellites* $w_{s}$ *(s = m + 1, m + 2, …, z) are added to SMRW, and the magnification is greater for *$\frac{1}{\prod_{m+1}^{z}w_{s}}$ *times.*

**Theorem** **2.** *For* $\frac{\sum_{s=1}^{m}w_{s}}{\prod_{s=1}^{m}w_{s}}$ *and* $\frac{\sum_{i=1}^{m}w_{i}}{\prod_{i=1}^{m}w_{i}}$*, if* $w_{s}\geq w_{i}$*, when s = i (i = 1, 2, …, m) after w_s_ and w_i_ are sorted from small to large, respectively,* $\frac{\sum_{s=1}^{m}w_{s}}{\prod_{s=1}^{m}w_{s}}$ *is less than or equal to* $\frac{\sum_{i=1}^{m}w_{i}}{\prod_{i=1}^{m}w_{i}}$.

The proofs of Theorems 1 and 2 are provided in Appendix A.

Consequently, Equation (14) can be rewritten:

(15) $$\ln\frac{\mathrm{ADOP}}{f_{1}}=\frac{1}{m-1}\left(\ln{\left(\mathrm{SMRW}\right)}^{\frac{1}{2}}+\ln{\left(\frac{C_{r}}{C_{h}}\right)}^{\frac{3}{2}}\right)$$

where $\ln\frac{\mathrm{ADOP}}{f_{1}}$ is called the Extend ADOP (E-ADOP), namely $\ln\frac{\mathrm{ADOP}}{f_{1}}=E–\mathrm{ADOP}$ which is proportional to ADOP.

Let $\ln{\left(\frac{C_{r}}{C_{h}}\right)}^{\frac{3}{2}}=\xi$, $\frac{1}{m-1}=\alpha$ and $\ln{\left(\mathrm{SMRW}\right)}^{\frac{1}{2}}=\beta$, then Equation (15) can be rewritten:

(16) E–ADOP=α(β+ξ)

Supposing ${\mathrm{SMRW}}^{\frac{1}{2}}=X$, $\frac{1}{m-1}=Y$ and ${\left(\frac{C_{r}}{C_{h}}\right)}^{\frac{3}{2}}=Z$, for ADOP, the $f_{2}$ is the multivariate power exponential function of X and Y and the $f_{3}$ is the exponential function of Y. In E-ADOP, the X and Y in $f_{2}$ are separated, namely the $f_{2}$ is broken down into α and β. α is equal to Y and β is the logarithmic function of X. In E-ADOP, the $f_{3}$ in ADOP is broken down into α and lnZ which is equal to ξ. Hence, the expression of E-ADOP makes the expression of ADOP which is the product of complex functions as the product of simple functions, which changes the dependence of ADOP and E-ADOP on m and SMRW. This change also makes E-ADOP expression more concise and intuitive and the expression of E-ADOP gives a clearer insight into the influences of number of satellites and SMRW on E-ADOP, which will make the theoretical analysis of E-ADOP more convenient than ADOP. The important property of E-ADOP is that the mean E-ADOP value can be predicted more accurately by the mean value of parameters through the expression of E-ADOP than through the ADOP expression for mean ADOP value, which is important for prediction of a new E-ADOP when some satellites are added based on Theorem 1 (see Proof 3 in Appendix A).

When the system and carrier phase are set, then $f_{1}$ and $\frac{C_{r}}{C_{h}}$ are confirmed, which makes ξ a constant and E-ADOP a dependent variable that is only related to ADOP and it is proportional to ADOP. The variables *α* and *β* are independent and they are related to the number of satellites and SMRW or satellite-elevation, respectively. Therefore, when the system and carrier phase are set, the E-ADOP is only the function of number of satellites and satellite elevation or SMRW.

#### 3.3.2. The Main Factors of SFSE E-ADOP for Short Baseline

Based on E-ADOP and Theorems 1 and 2, we analyze which factor, the number of satellites or SMRW, is dominant to control SFSE E-ADOP for short baseline.

Based on Table 1, for $\xi^{b}=14.36$ and $\xi^{g}=14.05$, the mean value of $\xi^{b}$ and $\xi^{g}$ is used in the next analysis, namely ξ=14.21. Superscript b and g represent BDS and GPS, respectively. Hence, Equation (16) becomes:

(17) E–ADOP=α(β+14.21)

The relations between E-ADOP, number of satellites and satellite elevation were analyzed.

**Figure 1:**
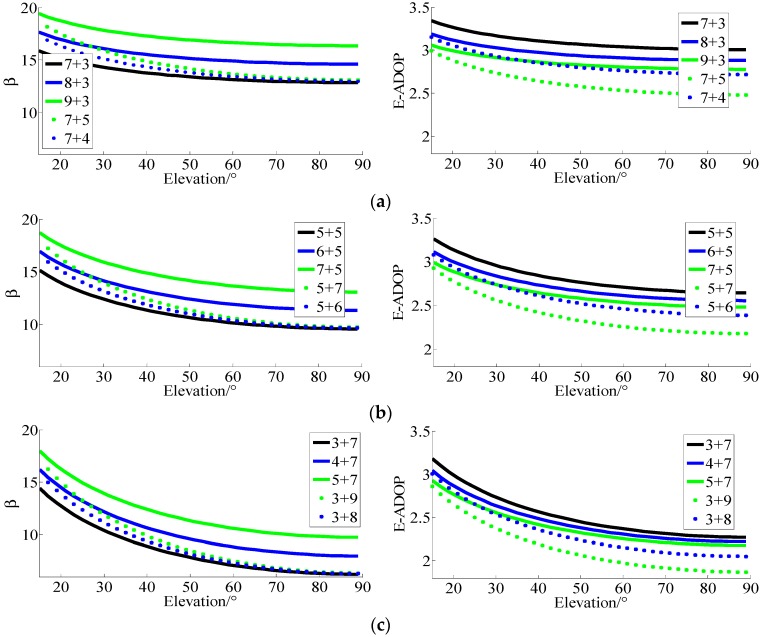
SMRW and E-ADOP of three groups of satellites. (**a**) The β and E-ADOP of (7 + 3) satellites. (**b**) The β and E-ADOP of (5 + 5) satellites. (**c**) The β and E-ADOP of (3 + 7) satellites.

The setting was as follows: one low- or high-elevation satellite or two low- or high-elevation satellites were, respectively, added to three groups of satellites to explore the influence of number of satellites and SMRW on E-ADOP. Each group of satellites consists of $n_{1}+n_{2}=10$ satellites which includes two kinds of satellites: one contains $n_{1}$ satellites whose elevations are all 10° and another contains $n_{2}$ same elevation satellites whose elevations are higher than those of $n_{1}$ satellites. Three groups of satellites were: (7 + 3), wherein the low-elevation satellites were majority, and results are shown in Figure 1a; (5 + 5), namely the low- and high-elevation satellites were equally distributed, and results are shown in Figure 1b; and (3 + 7), namely the high-elevation satellites were the majority, and results are shown in Figure 1c. In the result, the elevation of $n_{2}$ same elevation satellites was taken as x-axis, such as 15°, 20°, …, 90°.

In Figure 1, when a low-elevation satellite or two low-elevation satellites are added to three groups of satellites, *β* becomes larger and the more low-elevation satellites are added, the larger *β* is, which is consistent with Theorem 1. However, the corresponding E-ADOP is the opposite: the more low-elevation satellites are added, the smaller the E-ADOP is, even though *β* becomes larger. Therefore, based on Equation (17), the number of satellites is the dominant factor to control of E-ADOP when low-elevation satellites are added.

When a high-elevation satellite or two high-elevation satellites are added to three groups of ten satellites, β also becomes larger, which is consistent with the Theorem 1. The corresponding E-ADOP is the same as the E-ADOP when low-elevation satellites are added. Thus, based on Equation (17), the number of satellites is also the dominant factor to control of E-ADOP when high-elevation satellites are added.

However, differences in *β* between dotted lines and black solid line are much smaller than the differences between the black solid line and another color solid lines as the elevations of *n*_2_ satellites become larger or the proportion of high-elevation satellites becomes larger, which make the E-ADOP of dotted lines much smaller than those of the corresponding solid lines; furthermore, when the number of satellites is constant, the higher the number of high-elevation satellite is, the smaller *β* and E-ADOP are, in Figure 1. The above phenomena show that SMRW (high-elevation satellites) plays an important role in E-ADOP decrease.

Comparing green solid line and the corresponding blue dotted line in Figure 1, we can see that *β* of blue dotted line is much smaller than that of the corresponding green solid line, while the E-ADOP of blue dotted line is also smaller than that of the corresponding green solid line when the elevation of *n*_2_ satellites is larger than about 30°, although the number of satellites of green solid line is larger than corresponding blue dotted line. It is worth noting that the high-elevation satellite proportions of blue dotted line in Figure 1a–c are 36.36%, 54.55% and 72.73%, respectively; the high-elevation satellite proportions of green solid line in Figure 1a–c are 25%, 41.67% and 58.33%, respectively; and the differences in numbers of satellites between blue dotted line and the corresponding green solid line are equal to one, which means that in the case when the difference in number of satellites is not large, the larger the number of high-elevation satellites is, the smaller the E-ADOP value is. Moreover, in this circumstance, SMRW is more dominant than number of satellites in control of E-ADOP. In order to study the influence of a large number of high-elevation satellites on E-ADOP, two simulations were performed. In the first simulation, the total number of satellites was constant, but the proportion of high-elevation satellites was changed. In the second simulation, both the total number of satellites and the proportion of high-elevation satellite number were changed. The obtained results are as follows.

**Figure 2:**
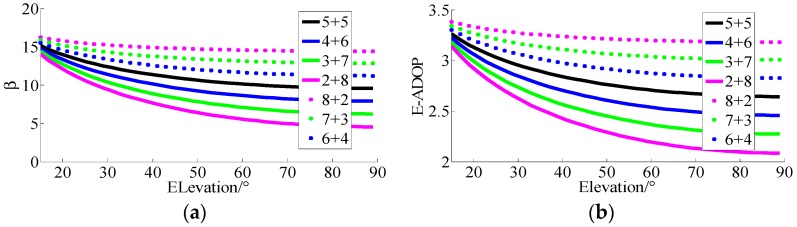
Factor *β* and E-ADOP of ten satellites for different elevations. (**a**) The β of ten satellites for different elevations. (**b**) The E-ADOP of ten satellites for different elevations.

For the first simulation, the results of *β* and E-ADOP for $n_{1}+n_{2}=10$ satellites are presented in Figure 2. In Figure 2, it can be seen that when the number of satellites is constant, the higher the number of high-elevation satellites is and the larger the elevations of n_2_ satellites are, the smaller β and E-ADOP are, which is consistent with Theorem 2. Consequently, when the number of satellites is constant, the greater proportion of high-elevation satellites is, the smaller the SMRW and E-ADOP are. According to Equation (17), in this circumstance, the SMRW is the dominant factor to control of E-ADOP.

**Figure 3:**
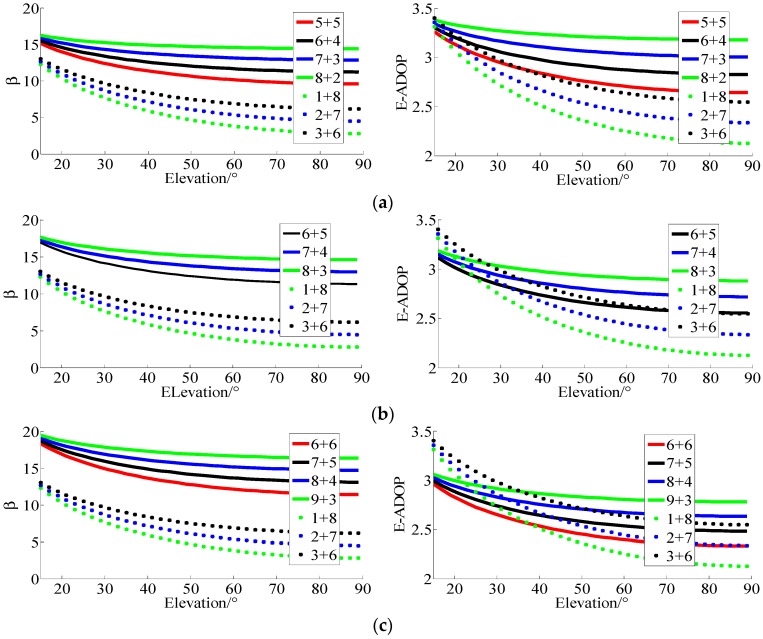
Factor *β* and E-ADOP of three groups of satellites for different elevations. (**a**) The β and E-ADOP of $n_{1}+n_{2}=10$ and $n_{1}+n_{2}=9$ satellites. (**b**) The β and E-ADOP of $n_{1}+n_{2}=11$ and $n_{1}+n_{2}=9$ satellites. (**c**) The β and E-ADOP of $n_{1}+n_{2}=12$ and $n_{1}+n_{2}=9$ satellites.

For the second simulation, the results of *β* and E-ADOP for $n_{1}+n_{2}$ satellites are presented in Figure 3. The $n_{1}+n_{2}$ has four groups. In the first three groups, $n_{1}+n_{2}=10,n_{1}+n_{2}=11,n_{1}+n_{2}=12$ are presented by solid lines which mean low-elevation satellites are the majority or low- and high-elevation satellites are equally distributed. The group $n_{1}+n_{2}=9$ is presented by the dotted line, which means high-elevation satellites are the majority, however the difference of number of satellites between the dotted line and solid line is not large. According to Figure 3a,b, although the numbers of satellites that correspond to the solid lines are larger than those that correspond to the dotted lines, all values of *β* of dotted lines are smaller than those of solid lines. At the same time, all values of E-ADOP of dotted lines are always smaller than those of solid lines when their corresponding elevations are larger than a certain level respectively, and, the larger the elevations of high-elevation satellites are, the more obvious the difference is, which is attributed to the large number of high-elevation satellites or SMRW. Therefore, according to Equation (17), we can conclude that, compared to the case where low-elevation satellites are the majority or low- and high-elevation satellites are equally distributed, when the high-elevation satellites are the majority, even if there are fewer satellites than in the first two cases mentioned above, the SMRW is the dominant factor to make E-ADOP smaller. However, the difference in number of satellites between solid line and dotted line is not large.

In Figure 3c, the results of *β* are the same as in Figure 3a,b. However, in Figure 3c, the E-ADOP of black dotted line is smaller than those of green and blue solid lines when the elevation is larger than a certain level, but it is larger than those of black and red solid lines. The E-ADOP of blue dotted line is smaller than those of green, blue and black solid lines when the elevation is larger than a certain level, however it is larger than that of red solid line. The E-ADOP of green dotted line is always smaller than those of solid lines when elevation is larger than a certain level. The numbers of high-elevation satellites that correspond to black, blue and green dotted lines are increased in turn. Thus, according to Figure 3a–c, the difference in number of satellites can be further expanded, as the proportion of high-elevation satellites becomes larger or the elevations of high-elevation satellites become larger.

According to the above analyses, few conclusions can be made: (1) the number of satellites mainly makes E-ADOP smaller when satellites are added; (2) when the number of satellites is constant, the greater the proportion of high-elevation satellites is, the smaller the E-ADOP is, i.e., the SMRW is the dominant factor that makes E-ADOP smaller; and (3) in contrast to the systems where the satellites with low-elevation are the majority or where low- and high-elevation satellites are equally distributed, in the systems where the high-elevation satellites are the majority, the SMRW makes E-ADOP smaller, even if there are fewer satellites than in the previous two cases, however, the difference in number of satellites should not be large and it can be further expanded as the proportion of high-elevation satellites becomes larger or the elevations of high-elevation satellites become larger. Conclusions (1)–(3) apply equally to ADOP.

#### 3.3.3. SFSE E-ADOP for Short Baseline Analyses of BDS, GPS and BDS/GPS

In [10], it was shown that success rate of BDS/GPS is higher than that of a single system and that success rate of BDS is higher than that of GPS, but it was not further studied which factor causes above phenomenon. This section analyzes the main factors for above phenomenon based on E-ADOP, namely the influences of number of satellites and SMRW or number of high-elevation satellites on E-ADOP are analyzed.

(1) Analysis of number of satellites in BDS/GPS, BDS and GPS

Firstly, the analysis on number of satellites for different cut-off elevation and baselines is obtained. The data were taken from the Hong Kong Base Station in March 2017 for baselines of 5 km, 10 km, and 15 km for BDS, GPS and BDS/GPS whose sampling interval was 30 s and the experiment lasted for 10 days. The obtained experimental results are shown in Table 2.

As can be seen in Table 2, for any cut-off elevation, the number of satellites is larger in BDS than in GPS; for instance, from cut-off 20° to 40°, for each cutoff-elevation, on average BDS has three satellites more than GPS and for cut-off 50° and 60°, the number of satellites in BDS is two times as large as that of GPS. In BDS, the proportion of high-elevation satellites whose elevation is larger than 35° is about 70%, which means the high-elevation satellites are the majority in Asia-Pacific Region, especially China, while, in GPS, that proportion is about 50%, which means the low- and high-elevation satellites are equally distributed. In comparison to the single system, for any cut-off elevation, BDS/GPS has much larger number of satellites than any of presented single systems. Moreover, in BDS/GPS, the proportion of high-elevation satellites is about 60%.

(2) SFSE E-ADOP analysis of GPS and BDS for short baseline

**Figure 4:**
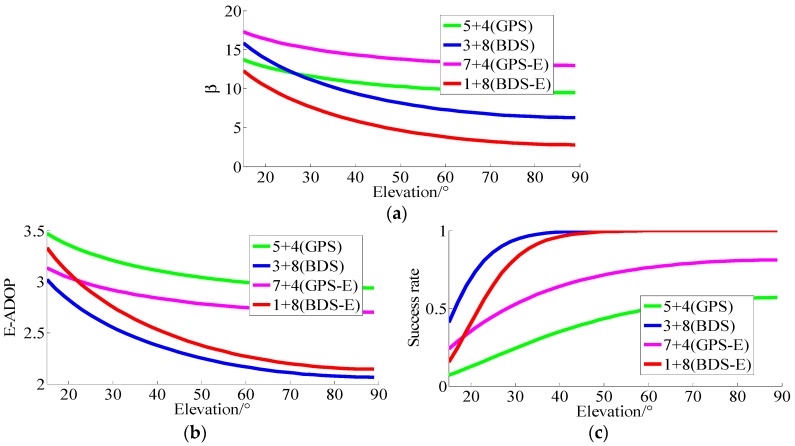
The *β*, E-ADOP and success rate of GPS, BDS, GPS-E and BDS-E. (**a**) The β of GPS, BDS, GPS-E and BDS-E. (**b**) The E-ADOP of GPS, BDS, GPS-E and BDS-E. (**c**) The success rate of GPS, BDS, GPS-E and BDS-E.

Teunissen et al. [10] indicated that the reason that the regional BDS has better single-frequency ambiguity resolution performance than GPS is caused mainly by its larger number of visible satellites. Based on above analyses, the large number of high-elevation satellites or SMRW can make *β* smaller and, according to Equation (16), the number of satellites and SMRW of BDS can make E-ADOP of BDS smaller compared to that of GPS. Determining which of these factors plays the major role is analyzed through two steps. In the first step, low-elevation satellites (elevation of 10° or similar) are added to GPS to make its number of satellites equal to BDS number of satellites, and this system is called GPS-extended (GPS-E). According to Conclusion (1), the number of satellites mainly makes E-ADOP of GPS-E smaller compared to that of GPS. The second step is based on adjustment of proportion of high-and low-elevation satellites in GPS-E in order to form BDS, and, according to Conclusion (2), a large number of high-elevation satellites or SMRW mainly makes E-ADOP of BDS smaller compared to E-ADOP of GPS-E. Based on Table 1, $\xi^{b}=14.36$ and $\xi^{g}=14.05$ in Equation (16). For BDS/GPS, the mean value of $\xi^{b}$ and $\xi^{g}$ was used, namely $\xi^{b/g}=14.21$. Superscript b and g represent BDS and GPS, respectively. Combined with Equation (16), the results are shown in Figure 4.

In Figure 4, *β* of BDS is smaller than *β* of GPS when the elevation of *n*_2_ satellites is higher than about 25° because of large number of high-elevation satellites in BDS. Based on the E-ADOP presented in Figure 4, when the elevation of $n_{2}$ satellites is higher than about 25° the E-ADOP of GPS-E is improved compared to GPS due to two added low-elevation satellites, but the difference is much smaller than the difference between GPS-E and BDS because the high- and low-elevation satellites proportion is adjusted. The larger the elevations of *n*_2_ satellites are, the more obvious the difference is. Therefore, Conclusion (4) can be made: a large number of high-elevation satellites or SMRW makes E-ADOP of BDS smaller than E-ADOP of GPS, which is consistent with Conclusion (3). In addition, in BDS-extended (BDS-E) the number of satellites is equal to the number of satellites in GPS, but the number of high-elevation satellites is equal as in BDS. According to the E-ADOP and success rate in Figure 4, a large number of high-elevation satellites of BDS-E can further make its E-ADOP smaller and success rate higher than those of GPS-E when the elevation of $n_{2}$ satellites is higher than about 25°, although its number of satellites is smaller than that of GPS-E, which is consistent with Conclusion (3). The improvement of E-ADOP from GPS to BDS-E is much larger than the improvement of E-ADOP from BDS-E to BDS, which further validates Conclusion (4). At the same time, large number of satellites in BDS further makes its E-ADOP smaller compared to E-ADOP of GPS.

(3) SFSE E-ADOP analysis of BDS/GPS and single system for short baseline

**Figure 5:**
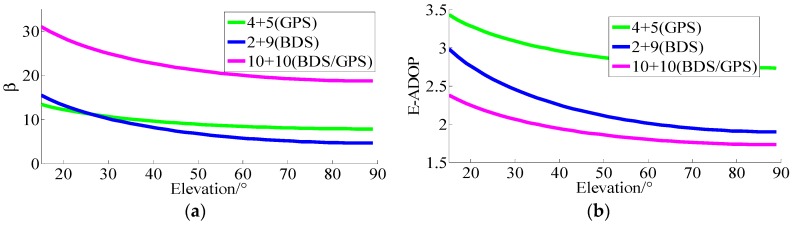
The *β* and E-ADOP of GPS, BDS and BDS/GPS. (**a**) The β of GPS, BDS and BDS/GPS. (**b**) The E-ADOP of GPS, BDS and BDS/GPS.

As already mentioned, three groups of $\left(n_{1}+n_{2}\right)$ satellites, wherein *n*_1_ denotes the number of low-elevation satellites and *n*_2_ denotes the number of high-elevation satellites, are used to study the influence of number of satellites and SMRW on the E-ADOP based on the E-ADOP theory. The results are shown in Figure 5.

In Figure 5, *β* of BDS/GPS is much larger than *β* of BDS and GPS, which is consistent with Theorem 1, while E-ADOP of BDS/GPS is smaller than E-ADOP of BDS and GPS. Based on Equation (16), Conclusion (5) can be made: a large number of satellites is the dominant factor to make E-ADOP of BDS/GPS smaller than E-ADOP of standalone BDS and GPS, which is consistent with Conclusion (1).

Conclusions (4) and (5) apply equally to ADOP.

## 4. Experimental Analyses of SFSE Positioning Performance Based on E-ADOP for Short Baseline

The SFSE positioning performance experiments for short baseline were conducted to analyze the SFSE positioning performance based on ADOP and E-ADOP from the perspective of the number of observed satellites, the number of high-elevation satellites, Position Dilution of Precision (PDOP), ambiguity fixed success rate and baseline vector precision and at the same time to verify the above conclusions. The first experiment was on BDS, GPS and BDS/GPS using the baseline data collected in clear sky conditions at Hong Kong Base Station. The second was the experiment on BDS, GPS and BDS/GPS using the data collected in complex conditions, such as multipath effect and urban canyon, in Xuzhou. In the results, the BDS-E which is similar to the one mentioned above and the GPS-E which is the same as to the one mentioned above were formed to analyze the performance based on Theorem 1 to study the influence of SMRW and number of satellites on ADOP and E-ADOP. In the experiments, the high-elevation denotes the elevation larger than 35° and the values presented in the following tables are the average values except the success rate value. The baseline vector accuracy was calculated by the baseline vector deviations of successful epochs in experiments. The baseline vectors calculated using the precise Hong Kong Base Station coordinates or calculated by professional software were taken as true values and the baseline vectors calculated using the real experimental data were called calculated values. The baseline vector error plots were determined by subtracting true values form corresponding calculated values of baseline vectors. The empirical ILS success rate $P_{s,E}$ is defined as [2,10,11]:

(18) $$P_{s,E}=\mathrm{Number}\;\mathrm{of}\;\mathrm{Successful}\;\mathrm{Epochs}/\mathrm{Total}\;\mathrm{Epochs}$$

The successful epoch means the epoch, in which the ambiguities are successfully fixed. The successfully fixed ambiguities must meet the following two conditions. The first is the successfully fixed ambiguities must comply with the ratio test, namely $\frac{R_{2}}{R_{1}}>\gamma$ [24]. $R_{1}$ and $R_{2}$ are the residual quadratics of “best integer candidate” and “second best integer candidate” of ambiguities, respectively. γ is a tolerance value and the value of γ is taken as 1.5 in these experiments. The second is that baseline vector error is within a certain range. When the ambiguities meet the above conditions for one epoch, we think the ambiguities are fixed successfully in this epoch.

In these experiments, B2 and L2 were used for positioning and according to results presented in Table 1, $f_{1}^{b}\approx 0.0142$, $f_{1}^{g}\approx 0.0145$, $f_{3}^{b}\approx {\left(120\right)}^{\frac{3}{m-1}}$ and $f_{3}^{g}\approx {\left(108\right)}^{\frac{3}{m-1}}$. For BDS/GPS, the average values of two system parameters were used, $f_{1}^{b/g}\approx 0.0144$, $f_{3}^{b/g}\approx {\left(114\right)}^{\frac{3}{m-2}}$ and $f_{2}^{b/g}={\left[\frac{\sum_{s=1}^{m}w_{s}}{\prod_{s=1}^{m}w_{s}}\right]}^{\frac{1}{2\left(m-2\right)}}$. Superscript b and g represent BDS and GPS, respectively.

### 4.1. Experiments in Clear Sky Conditions

**Figure 6:**
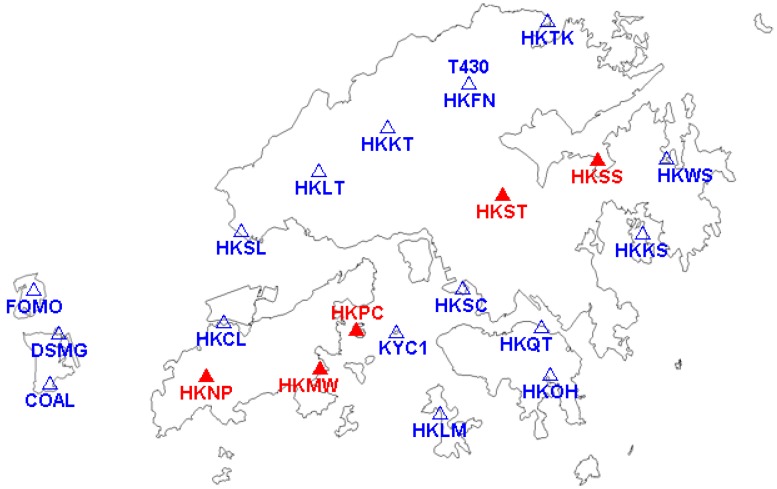
Distribution of Hong Kong Base Stations.

In this paper, the experiments on SFSE ambiguity resolution of GPS, BDS and BDS/GPS were performed using the data for baseline of 5 km, 10 km and 15 km from the Hong Kong Base Station in 2017 whose sampling intervals were 1 s. The distribution of Hong Kong Base Stations is as shown in Figure 6. The red stations were used in experiments and the distance between HKPC and HKMW was about 5 km, the distance between HKST and HKSS was about 10 km, and the distance between HKNP and HKPC was about 15 km. For a short baseline, both ionospheric and the tropospheric delay errors can be eliminated theoretically by the double-difference model, and in order to reduce the influences of these errors the two-day data from 20:00 to 8:00 the next day were adopted.

#### 4.1.1. Experiments for 5 km Baseline

**Figure 7:**
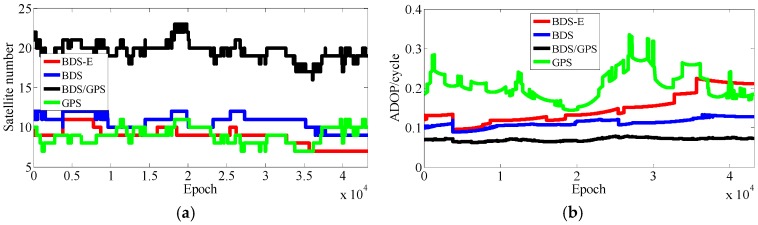
Satellite number and ADOP of four systems for group A. (**a**) The satellite number of four systems for group A. (**b**) The ADOP of four systems for group A.

**Figure 8:**
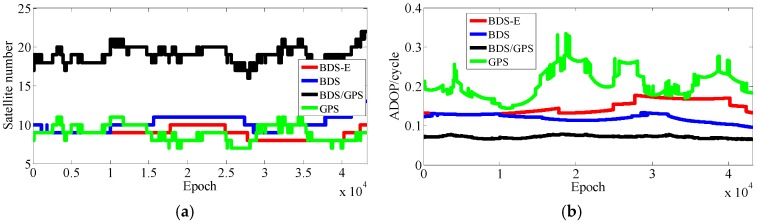
Satellite number and ADOP of four systems for group B. (**a**) The satellite number of four systems for group B. (**b**) The ADOP of four systems for group B.

**Figure 9:**
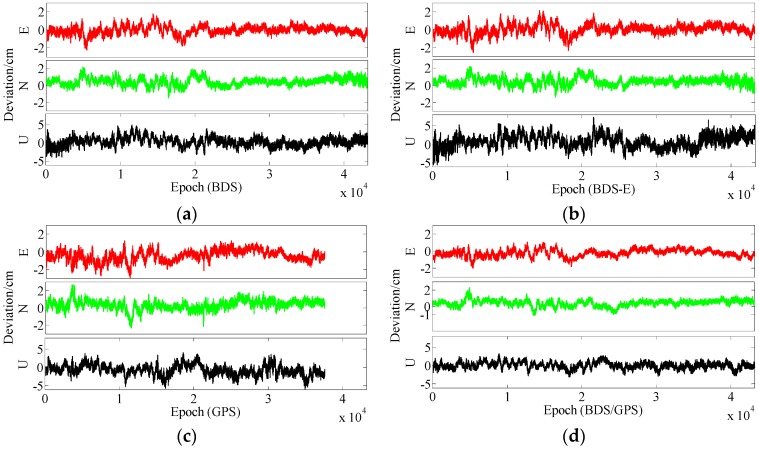
East (E), North (N) and Up (U) deviations of four systems for group A. (**a**) ENU deviations of BDS for group A. (**b**) ENU deviations of BDS-E for group A. (**c**) ENU deviations of GPS for group A. (**d**) ENU deviations of BDS/GPS for group A.

**Figure 10:**
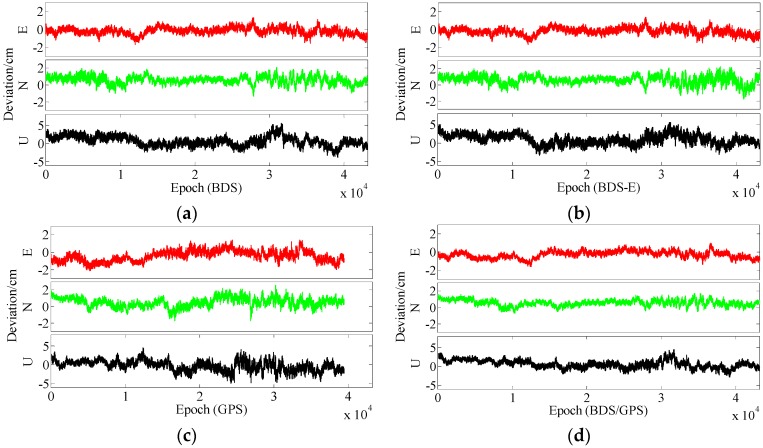
ENU deviations of four systems for group B. (**a**) ENU deviations of BDS for group B. (**b**) ENU deviations of BDS-E for group B. (**c**) ENU deviations of GPS for group B. **(d**) ENU deviations of BDS/GPS for group B.

The results of experiments for 5 km baseline using data collected in two days data are shown as Table 3 and Figure 7, Figure 8, Figure 9 and Figure 10. In the following, if there is no clear statement, the success rate is $P_{s,E}$.

As presented in Table 3, BDS/GPS has the highest success rate, and then follow BDS and BDS-E, while GPS has the worst success rate, whose order is the same as ADOP and E-ADOP. The success rates of BDS/GPS, BDS and BDS-E are larger than 99.9%, while GPS success rate is only about 90% even it has the same number of satellites as BDS-E; moreover, all success rates are higher than their corresponding $P_{s,\mathrm{IB}}$. Based on the results in Table 3 and Figure 7 and Figure 8, the ADOP of BDS/GPS is always smaller than 0.1 and its mean value is about 0.07, the ADOP of BDS is smaller than 0.12 but it is always larger than ADOP of BDS/GPS, whose mean value is 0.115. The ADOP of GPS fluctuates considerably and it is above ADOP of BDS, whose mean value is 0.206, and the ADOP of BDS-E whose mean value is 0.147 is between BDS ADOP and GPS ADOP. In Table 3, *β* and E-ADOP/ADOP of BDS-E are both smaller than those of GPS because of the large number of high-elevation satellites which is consistent with Theorem 2 and Conclusion (2). In BDS-E and GPS, based on Equation (16), SMRW is the dominant factor to control E-ADOP and ADOP, which is consistent with Conclusion (2). Based on the Theorem 1, if two low-elevation satellites (whose elevation is about 10°) and one low-elevation satellite are added to GPS of Groups A and B, respectively, to make their numbers of satellites almost equal to the number of satellites in BDS, namely GPS-E, the ${\mathrm{SMRW}}^{\frac{1}{2}}$ of GPS of Groups A and B will be expanded at least 33.163 and 5.758 times, respectively. These changes will make the E-ADOP of GPS-E of Groups A and B larger than 2.45 and 2.52, respectively, and the corresponding ADOP larger than 0.168 and 0.180, respectively, all of which will still be larger than E-ADOP and ADOP of BDS-E. That means the improvements of E-ADOP and ADOP of BDS-E compared to GPS, caused by adjustment of proportion of the high- and low-elevation satellites, are larger than those of GPS-E compared to GPS because of added low-elevation satellites, although the number of satellites in BDS-E is smaller than in GPS-E. Thus, in this circumstance, the SMRW is dominant factor to control E-ADOP and ADOP, which is consistent with Conclusion (3). In Table 3, the maximum improvements of E-ADOP of GPS-E compared to GPS, caused by added low-elevation satellites, are about 0.18 and 0.10 for Groups A and B, respectively. Mentioned improvements are smaller than those of GPS-E compared to BDS because of adjustment of proportion of the high- and low-elevation satellites which are about 0.41 and 0.46 for Groups A and B, respectively. Further, ADOP is the same as E-ADOP. In summary, the large number of high-elevation satellites or SMRW is the dominant factor to make the E-ADOP and ADOP of BDS smaller and the success rate of BDS larger than those of GPS, which is consistent with Conclusion (4). The large number of satellites in BDS further makes its E-ADOP and ADOP smaller than that of GPS. Comparing the number of satellites, *β*, E-ADOP and ADOP of BDS, GPS and BDS/GPS presented in Table 3, it can be seen that in BDS/GPS the number of satellites and *β* are much larger than in GPS and BDS, while E-ADOP and ADOP of BDS/GPS are much smaller than in BDS and GPS. Hence, based on Equation (16), a large number of satellites is the dominant factor to make E-ADOP and ADOP of BDS/GPS smaller than those of BDS and GPS, which is consistent with Conclusion (5).

In terms of the baseline vector accuracy, according to Figure 9 and Figure 10, the maximal errors in E and N directions of four systems are all about 2 cm. In U direction, the maximal error is larger, and it is about 5 cm. From the aspect of baseline accuracy, in Table 3, for Group B the order of baseline accuracy from high to low in turn is BDS/GPS, GPS, BDS and BDS-E, GPS and BDS are similar in baseline accuracy, which is the same as for PDOP. For Group A, the order of baseline accuracy from high to low in turn is BDS/GPS, BDS, GPS and BDS-E. The baseline accuracy of BDS is higher than that of GPS which is not consistent with GPS PDOP and BDS PDOP. The reason may be as follows. As mentioned above, the observation accuracy of high-elevation satellite is higher than that of low-elevation satellite because the high-elevation satellite are less affected by multipath and ionosphere and a large proportion of high-elevation satellites can improve system reliability. The high-elevation satellites of BDS in Group A were the majority, while for GPS low- and high-elevation satellites were equally distributed. The much observations of low-elevation satellites of GPS in Group A might be affected by the multipath which made the accuracy of these observations lower than the accuracy of these observations of BDS or the ionospheric errors were not completely eliminated due to much low-elevation satellites in GPS, which made the positioning accuracy of GPS lower than that of BDS. The baseline accuracies are all about 1.2 cm and the difference between them is several millimeters.

#### 4.1.2. Experiments for 10 km Baseline

**Figure 11:**
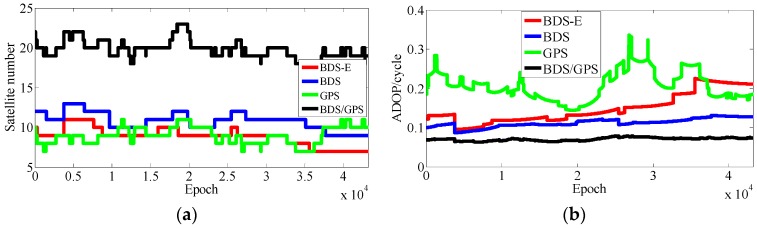
Satellite number and ADOP of four systems for group A. (**a**) The satellite number of four systems for group A. (**b**) The ADOP of four systems for group A.

**Figure 12:**
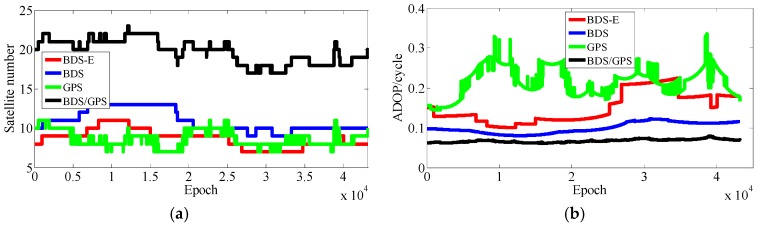
Satellite number and ADOP of four systems for group B. (**a**) The satellite number of four systems for group B. (**b**) The ADOP of four systems for group B.

**Figure 13:**
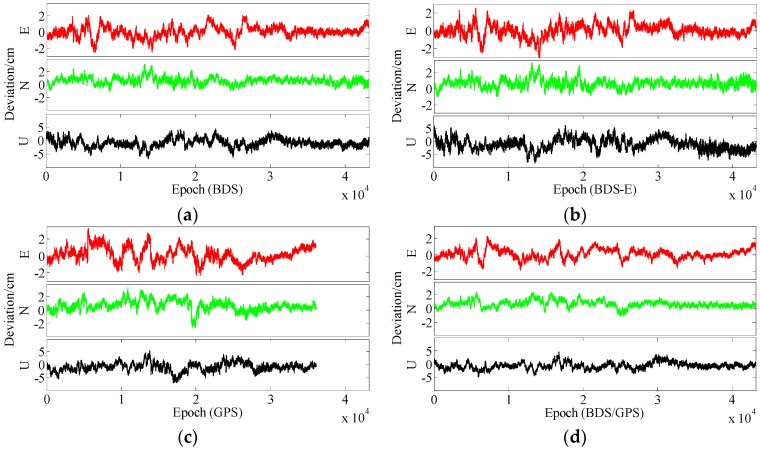
ENU deviations of four systems for group A. (**a**) ENU deviations of BDS for group A. (**b**) ENU deviations of BDS-E for group A. (**c**) ENU deviations of GPS for group A. (**d**) ENU deviations of BDS/GPS for group A.

**Figure 14:**
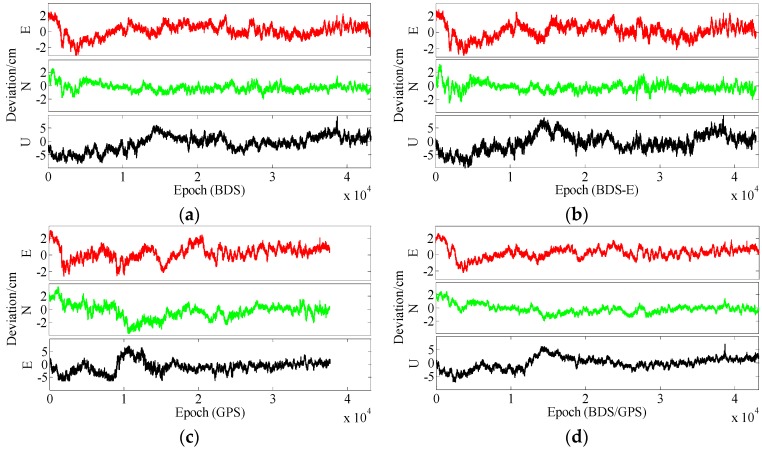
ENU deviations of four systems for group B. (**a**) ENU deviations of BDS for group B. (**b**) ENU deviations of BDS-E for group B. (**c**) ENU deviations of GPS for group B. (**d**) ENU deviations of BDS/GPS for group B.

The results of experiments for 10 km baseline using two-day data are shown in Table 4 and Figure 11, Figure 12, Figure 13 and Figure 14.

In Table 4, for Groups A and B, the success rate, ADOP and E-ADOP are the same as in the previous experiments (the experiments for 5-km baseline), respectively. The success rates of BDS/GPS and BDS are larger than 99.99%, the success rate of BDS-E is larger than 99%, and the success rate of GPS is only about 85% although GPS has more satellites than BDS-E; in addition, the success rates are all larger than corresponding $P_{s,\mathrm{IB}}$. Based on the results presented in Table 4 and Figure 11 and Figure 12, the ADOP distribution is the same as in the 5 km baseline experiments, and the mean values of ADOP of BDS/GPS, BDS, BDS-E and GPS are 0.069, 0.106, 0.149 and 0.21, respectively. Based on Equation (16) and comparing the number of satellites, *β*, E-ADOP/ADOP of BDS-E and GPS presented in Table 4, the SMRW is the dominant factor to make the E-ADOP and ADOP of BDS-E smaller than those of GPS, which is consistent with Conclusion (2).

Similar to the 5 km experiments, if two low-elevation satellites are added to GPS of Groups A and B, respectively, to make their numbers of satellites equal to that of BDS, namely GPS-E, based on Theorem 1, the ${\mathrm{SMRW}}^{\frac{1}{2}}$ of GPS will be expanded at least 33.163 times, which will make E-ADOP of GPS-E larger than 2.45 and the corresponding ADOP larger than 0.168 for Groups A and B, respectively, all of which are still larger than E-ADOP and ADOP of BDS-E. That means the improvements of E-ADOP and ADOP of BDS-E compared to GPS, caused by adjustment of proportion of the high- and low-elevation satellites, are larger than those of GPS-E compared to GPS because of added low-elevation satellites, although the number of satellites in BDS-E is smaller than in GPS-E. In this case, the SMRW is the dominant factor to control E-ADOP and ADOP, which is consistent with Conclusion (3). In Table 4, the maximum improved E-ADOP of GPS-E compared to GPS due to added low-elevation satellites is about 0.20 for both Groups A and B, and it is smaller than the improvement of E-ADOP of BDS compared to GPS-E due to adjustment of proportion of high- and low-elevation satellites, which is about 0.41. In addition, the ADOP is the same as the E-ADOP. In summary, the conclusion is the same as for the 5 km experiments: the large number of high-elevation satellites or SMRW is the dominant factor to make the E-ADOP and ADOP of BDS smaller and the success rate of BDS larger than those of GPS, which is consistent with Conclusion (4). The large number of satellites in BDS can further make its E-ADOP and ADOP smaller than that of GPS. Similar to the 5 km experiments, if we compare the number of satellites, *β* and E-ADOP/ADOP of BDS, GPS and BDS/GPS presented in Table 4, we can conclude that a large number of satellites makes E-ADOP and ADOP of BDS/GPS smaller than those of BDS and GPS, which is consistent with Conclusion (5).

In terms of the baseline vector accuracy, according to Figure 13 and Figure 14, the maximal errors in E and N directions for four systems are all about 3 cm, while the maximal errors in U direction are larger, and for BDS/GPS it is 5.5 cm, for GPS it is 6 cm, for BDS it is 7 cm, and for BDS-E it is 7.5 cm. From the aspect of baseline accuracy, the order of baseline accuracy from high to low in turn in Table 4 is BDS/GPS, GPS, BDS and BDS-E, and GPS and BDS are similar in baseline accuracy, which is the same as for PDOP. The baseline accuracies of BDS/GPS, GPS and BDS reached about 2 cm and the baseline accuracy of BDS-E reached 2.5 cm, while the difference between each other is not larger than 1 cm.

#### 4.1.3. Experiments for 15 km Baseline

**Figure 15:**
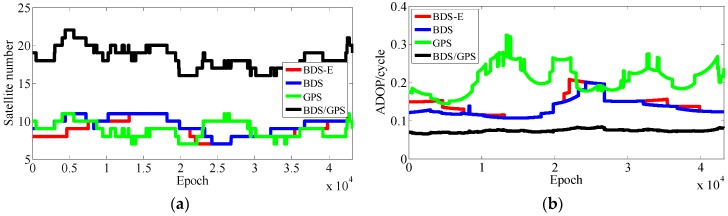
Satellite number and ADOP of four systems for group A. (**a**) The satellite number of four systems for group A. (**b**) The ADOP of four systems for group A.

**Figure 16:**
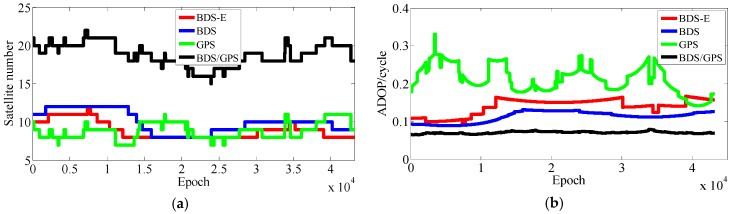
Satellite number and ADOP of four systems for group B. (**a**) The satellite number of four systems for group B. (**b**) The ADOP of four systems for group B.

**Figure 17:**
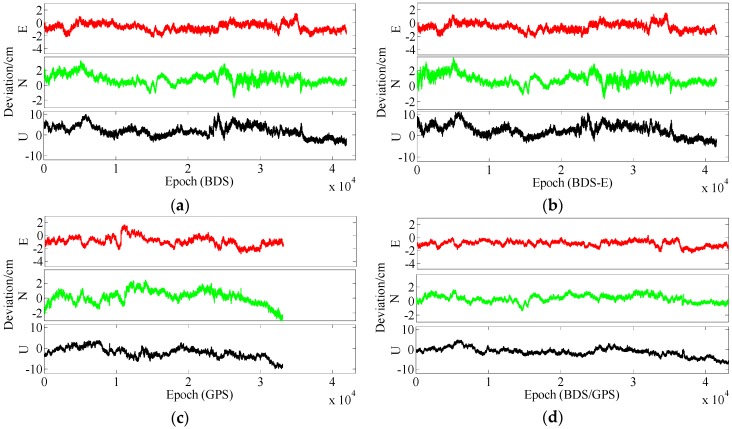
ENU deviations of four systems for group A. (**a**) ENU deviations of BDS for group A. (**b**) ENU deviations of BDS-E for group A. (**c**) ENU deviations of GPS for group A. (**d**) ENU deviations of BDS/GPS for group A.

**Figure 18:**
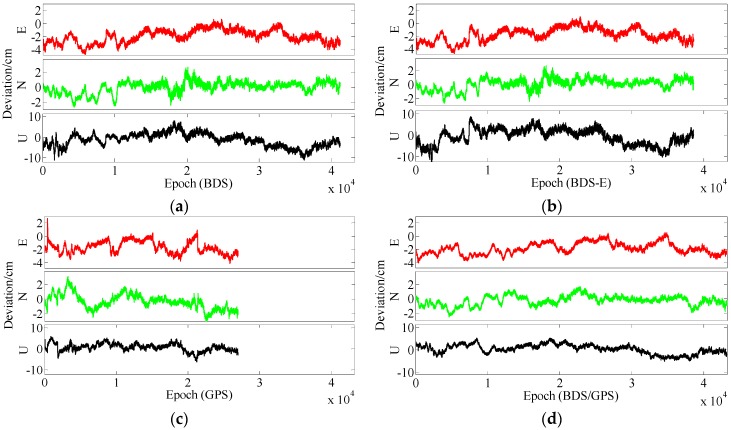
ENU deviations of four systems for group B. (**a**) ENU deviations of BDS for group B. (**b**) ENU deviations of BDS-E for group B. (**c**) ENU deviations of GPS for group B. (**d**) ENU deviations of BDS/GPS for group B.

The results of experiments for 15 km baseline using two day data are shown in Table 5 and Figure 15, Figure 16, Figure 17 and Figure 18.

According to the results presented in Table 5, the success rate, ADOP and E-ADOP of Groups A and B have the same trend as in previous two experiments. The success rate of BDS/GPS is larger than 99.99%, the success rates of BDS, BDS-E, and GPS are about 97%, 93% and 70%, respectively, although GPS has the same number of satellites as BDS-E; in addition, the success rates are all smaller than their corresponding $P_{s,\mathrm{IB}}$ except in the case of BDS/GPS, wherein the difference might be caused by the ionospheric error that was not eliminated by double-difference observation for 15 km baseline. According to Table 5 and Figure 15 and Figure 16, ADOP changes the same as in previous experiments and the mean values of ADOP of BDS/GPS, BDS, BDS-E and GPS are about 0.072, 0.123, 0.140 and 0.21, respectively. Comparing the number of satellites, *β* and E-ADOP/ADOP of BDS-E and GPS presented in Table 5, and based on Equation (16), the SMRW is dominant in control of E-ADOP/ADOP for BDS-E and GPS, which is consistent with Conclusion (2).

Similar to the previous experiments, which were conducted for 5 km and 10 km baselines, if one low-elevation satellite is added to GPS of both Groups A and B in order to make their numbers of satellites equal to that of BDS, namely GPS-E, based on Theorem 1, the ${\mathrm{SMRW}}^{\frac{1}{2}}$ of GPS will be expanded at least 5.758 times. This change will make the E-ADOP of GPS-E larger than 2.51 and the corresponding ADOP larger than 0.178 for Groups A and B, respectively, which will be still all larger than E-ADOP and ADOP of BDS-E. That means the improvements of E-ADOP and ADOP of BDS-E compared to GPS, due to adjustment of proportion of the high- and low-elevation satellites, are larger than those of GPS-E compared to GPS due to added low-elevation satellites, although the number of satellites in BDS-E is smaller than in GPS-E. Therefore, in this case, the SMRW is dominant in control of E-ADOP and ADOP, which is consistent with Conclusion (3). In Table 5, the maximal improvement of E-ADOP from GPS to GPS-E due to added low-elevation satellites is about 0.13 for both Groups A and B, and it is smaller than the improvement from GPS-E to BDS caused by adjustment of proportion of the high- and low-elevation satellites proportion, which is about 0.31. Nevertheless, the ADOP is same as the E-ADOP. In summary, the conclusion is the same as for the previous experiments: the large number of high-elevation satellites mainly makes the BDS have smaller E-ADOP and ADOP and higher success rate, which is consistent with Conclusion (4). The large number of satellites in BDS can further make its E-ADOP and ADOP smaller than that of GPS. Similar to 5 km and 10 km experiments, here we also compare the number of satellites, *β* and E-ADOP or ADOP of BDS, GPS and BDS/GPS presented in Table 5, and we can conclude that a large number of satellites makes the E-ADOP and ADOP of BDS/GPS smaller than those of BDS and GPS, which is consistent with Conclusion (5).

From the aspect of the baseline vector accuracy, in Figure 17 and Figure 18, the maximal errors in E and N directions for four systems are all about 4 cm, while the maximal errors in U direction are larger, in BDS/GPS and GPS they are lower than 10 cm, and in BDS and BDS-E they are about 10 cm. Furthermore, the order of baseline accuracy from high to low in turn is BDS/GPS, GPS, BDS and BDS-E, and GPS and BDS are similar in baseline accuracy, which is the same as for PDOP. The baseline accuracies of BDS/GPS, GPS and BDS are about 2.8 cm and the baseline accuracy of BDS-E is about 3.5 cm, and the difference between each other is not larger than 1 cm.

### 4.2. Experiments in Complex Sky Conditions

The data used in the experiments in clear sky conditions were collected in Xuzhou. The complex conditions include effects of multipath and urban canyon. The settings in these experiments were the same as in the experiments conducted in clear sky conditions.

#### 4.2.1. Low Dynamic Experiment in Urban Canyon Conditions

**Figure 19:**
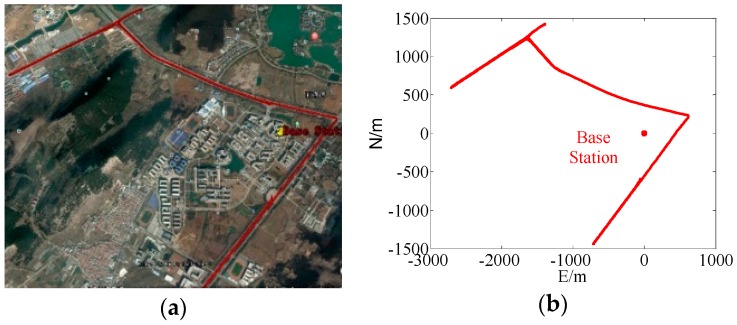
Movement trajectory. (**a**) The actual movement trajectory. (**b**) The simulated movement trajectory.

**Figure 20:**
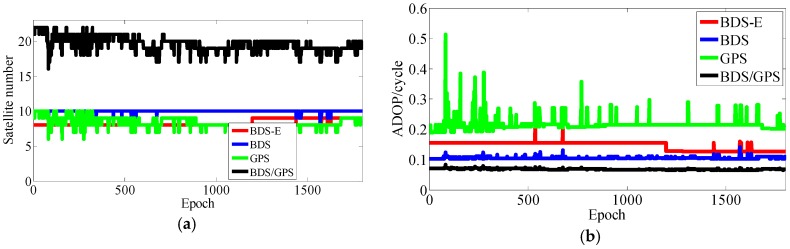
Satellite number and ADOP of four systems. (**a**) The satellite number of four systems. (**b**) The ADOP of four systems.

**Figure 21:**
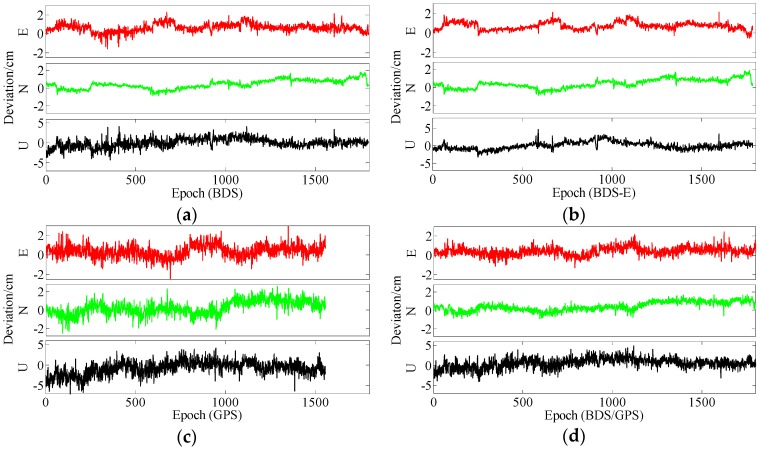
ENU deviations of four systems. (**a**) ENU deviations of BDS. (**b**) ENU deviations of BDS-E. (**c**) ENU deviations of GPS. (**d**) ENU deviations of BDS/GPS.

In the experiment, the coverage area of low dynamic data in urban canyon conditions was about 3 km^2^, its sampling interval was 1 s and the experiment lasted for 0.5 h. The coverage range was small because the baseline length of GPS can calculate was shorter than 5 km, according to the 5 km experiments. The results are shown in Table 6 and Figure 19, Figure 20 and Figure 21.

In Table 6, the success rate, ADOP and E-ADOP from high to low in turn are the same as for the experiments in clear sky conditions, respectively. The success rate of BDS/GPS is 100.00%, the success rate of both BDS and BDS-E is larger than 99%, and the success rate of GPS is about 86% although GPS has the same number of satellites as BDS-E; and all success rates are larger than their corresponding $P_{s,\mathrm{IB}}$. Based on results in Table 6 and Figure 20, the ADOP follows the same trend as in the previous experiments conducted in clear sky conditions and the values of ADOP of BDS/GPS, BDS, BDS-E and GPS are 0.069, 0.111, 0.146 and 0.217, respectively. Comparing the number of satellites, *β* and E-ADOP/ADOP of BDS-E and GPS presented in Table 6, and based on Equation (16), the conclusion that the SMRW is the dominant factor to control of E-ADOP and ADOP of BDS-E and GPS can be gotten, which is consistent with Conclusion (2).

Similar to the experiments in clear sky conditions, if two low-elevation satellites are added to GPS to make it have the same number of satellites as BDS, namely GPS-E, based on Theorem 1, the ${\mathrm{SMRW}}^{\frac{1}{2}}$ of GPS will be expanded at least 33.163 times. This change will make the E-ADOP of GPS-E larger than 2.48 and the corresponding ADOP larger than 0.173, which will be still larger than E-ADOP and ADOP of BDS-E. That means the improvements of E-ADOP and ADOP of BDS-E compared to GPS, caused by adjustment of proportion of the high- and low-elevation satellites, are larger than those of GPS-E compared to GPS caused by added low-elevation satellites, although the number of satellites in BDS-E is smaller than in GPS-E. Thus, in this case, the SMRW is the dominant factor in control of E-ADOP and ADOP, which is consistent with Conclusion (3). In Table 6, the maximal improvement of E-ADOP from GPS to GPS-E due to added a little low-elevation satellites is about 0.25, and it is smaller than the improvement from GPS-E to BDS due to adjustment of the proportion of high- and low-elevation satellites, which is equal to 0.43. In addition, the ADOP is the same as the E-ADOP. In summary, the conclusion is the same as for the experiments in clear sky conditions: the large number of high-elevation satellites or SMRW is the dominant factor to make the E-ADOP and ADOP of BDS smaller and the success rate of BDS larger than those of GPS, which is consistent with Conclusion (4). The large number of satellites in BDS can further make its E-ADOP and ADOP smaller than those of GPS. Similar to the experiments in clear sky conditions, we compare the number of satellites, *β* and E-ADOP/ADOP of BDS, GPS and BDS/GPS presented in Table 6, and we conclude that a large number of satellites makes E-ADOP and ADOP of BDS/GPS smaller than those of BDS and GPS, which is consistent with Conclusion (5).

#### 4.2.2. Multipath Effect Experiment

**Figure 22:**
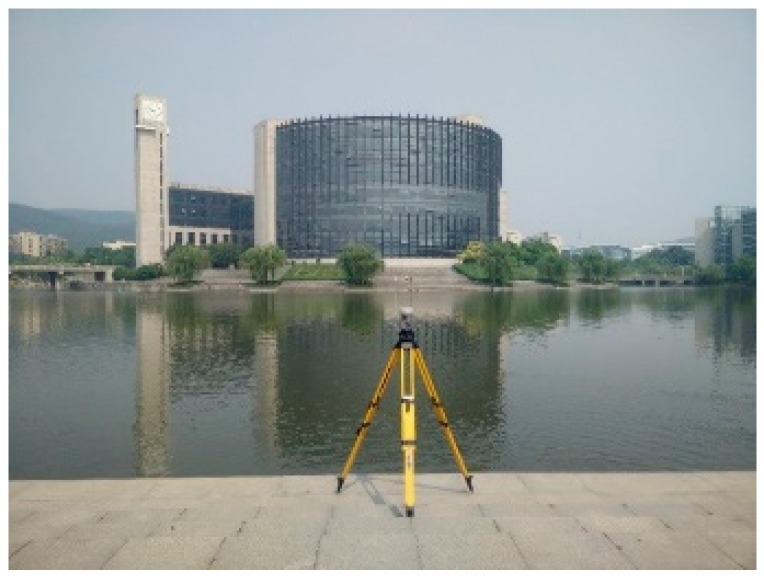
Multipath effect experimental setup.

**Figure 23:**
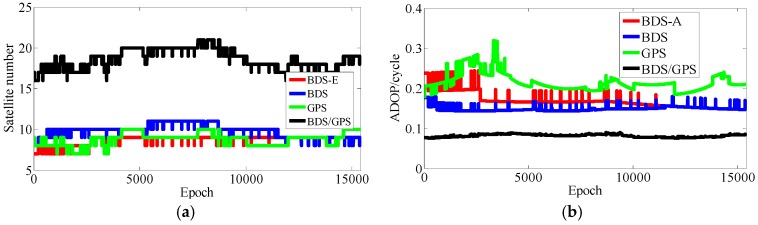
Satellite number and ADOP of four systems. (**a**) The satellite number of four systems. (**b**) The ADOP of four systems.

**Figure 24:**
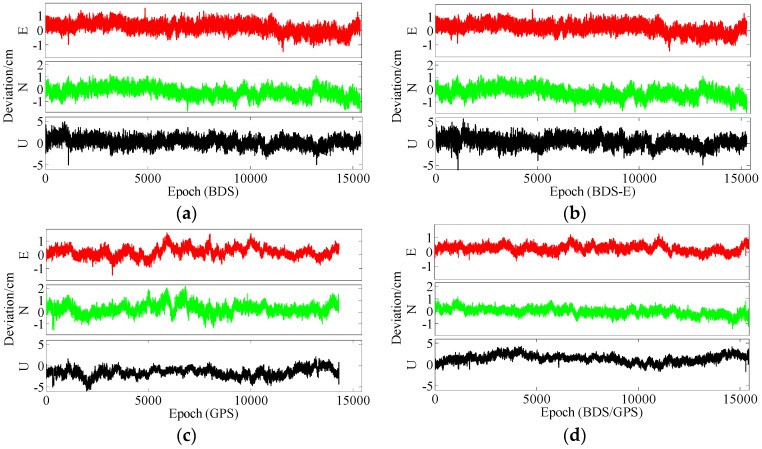
ENU deviations of four systems. (**a**) ENU deviations of BDS. (**b**) ENU deviations of BDS-E. (**c**) ENU deviations of GPS. (**d**) ENU deviations of BDS/GPS.

The baseline length in the multipath effect experiment was about 500 m, the sampling interval was 1 s and the experiment lasted for 4.3 h. The obtained results are shown in Table 7 and Figure 22, Figure 23 and Figure 24.

In Table 7, the trends of success rate, ADOP and E-ADOP are the same as in the experiments conducted in clear sky conditions. The success rate of BDS/GPS is 100.00%, the success rate of BDS is larger than 99%, the success rate of BDS-E is about 99%, and the success rate of GPS is about 93% although the number of satellites in GPS is the same as in BDS-E; in addition, all success rates are larger than their corresponding $P_{s,\mathrm{IB}}$. According to the results in Table 7 and Figure 23, the ADOP trend is the same as in the experiments conducted in clear sky conditions and ADOP of BDS/GPS is 0.073, while the values of ADOP of BDS, BDS-E, and GPS are about 0.146, 0.168 and 0.212, respectively. If we compare the number of satellites, *β* and E-ADOP/ADOP of BDS-E and GPS presented in Table 7, and based on Equation (16), we can conclude that SMRW is the dominant factor in control of E-ADOP and ADOP in both BDS-E and GPS, which is consistent with Conclusion (2).

As in the experiments in clear sky conditions, if one low-elevation satellite is added to GPS to make its number of satellites equal to that of BDS, namely GPS-E, based on Theorem 1, the ${\mathrm{SMRW}}^{\frac{1}{2}}$ of GPS will be expanded at least 5.758 times. This change will make E-ADOP of GPS-E larger than 2.56 and the corresponding ADOP larger than 0.188, which will still be larger than E-ADOP and ADOP of BDS-E. That means the improvements of E-ADOP and ADOP of BDS-E compared to GPS, caused by adjustment of proportion of the high- and low-elevation satellites, are larger than those of GPS-E compared to GPS caused by added low-elevation satellites, although the number of satellites in BDS-E is smaller than in GPS-E. Hence, in this case, the SMRW is the dominant to control E-ADOP and ADOP, which is consistent with Conclusion (3). In Table 7, the maximal improvement of E-ADOP from GPS to GPS-E due to added low-elevation satellites is about 0.11, which is smaller than the improvement from GPS-E to BDS due to adjustment of proportion of the high- and low-elevation satellites, which is equal to 0.22. Moreover, the ADOP is the same as the E-ADOP. In summary, the conclusion is the same as for the experiments in clear sky conditions: the large number of high-elevation satellites or SMRW is the dominant factor to make the E-ADOP and ADOP of BDS smaller and the success rate of BDS larger than those of GPS, which is consistent with Conclusion (4). The large number of satellites in BDS can further make its E-ADOP and ADOP smaller than those of GPS. As for the experiments in clear sky conditions, if we compare the number of satellites, *β* and E-ADOP/ADOP of BDS, GPS and BDS/GPS presented in Table 7, we can see that large number of satellites makes the E-ADOP and ADOP of BDS/GPS smaller than those of BDS and GPS, which is consistent with Conclusion (5).

From the aspect of baseline accuracy, in Table 6 and Table 7, the order of baseline accuracy from high to low in turn is BDS, BDS-E, BDS/GPS and GPS, and the BDS and BDS-E are similar in baseline accuracy, which is not consistent with PDOP. The main reason for this phenomenon is as follows. In GPS, there are many low-elevation satellites, and the signal of low-elevation satellites might be influenced by the multipath effect or be blocked in complex sky conditions, which could cause poor signal quality and result in a low positioning accuracy. When BDS and GPS are combined, the number of high-elevation satellites becomes larger which can improve the overall signal quality, while the number of low-elevation satellites also becomes larger and the positioning accuracy can be influenced by low-elevation satellites with poor signal quality; thus, the baseline accuracy of BDS/GPS is worse than those of BDS and BDS-E, but better than that of GPS. This is also the reason why the baseline accuracy in complex sky conditions is not higher than that in 5 km experiments. The baseline accuracy of four systems reached 1.5 cm in the experiments with urban canyon and multipath effect, and the difference between these accuracies of different systems is about several millimeters. In Figure 21, the maximal errors in E and N directions of four systems are about 2 cm, and the maximal errors in U direction of BDS/GPS, BDS and BDS-E are about 5 cm, while the error of GPS in U direction is larger, and it is about 7 cm. In Figure 24, the maximal errors in E and N directions of BDS/GPS, BDS and BDS-E are about 1 cm, and the maximal errors of GPS are about 2 cm. The maximal errors in U direction of BDS/GPS, BDS and BDS-E are about 5 cm, and the maximal error of GPS is about 6 cm.

According to the above analyses, the conclusions which are obtained by empirical analyses and experiments with different short baselines in Asia-Pacific Region are consistent with the conclusions obtained by the theoretical analyses. We can also see that the improvement of E-ADOP and ADOP from GPS to BDS-E, caused by adjustment of proportion of high- and low-elevation satellites, is larger than the improvement of E-ADOP and ADOP from BDS-E to BDS due to added low-elevation satellites, which further validates Conclusion (4).

## 5. Conclusions

The SFSE positioning has always been the hot spot of the high-precision GNSS positioning due to its low success rate of ambiguity resolution. The ADOP is a well-known scalar measure which can be used to infer the strength of GNSS model for carrier phase ambiguity resolution. Traditional ADOP expression is complicated; thus, through theoretical deduction, the SFSE E-ADOP, with SMRW and two theorems for short baseline, is developed. That simplifies the ADOP expression and makes theoretical analysis of E-ADOP more convenient than that of ADOP and through that the mean E-ADOP value can be predicted more accurately by the mean value of parameters through the expression of E-ADOP than through the ADOP expression for mean ADOP value. E-ADOP gives a clear insight into the influence of number of satellites and SMRW or the number of high-elevation satellites on ADOP and reveals that the number of satellites and SMRW or the number of high-elevation satellites are important for ADOP. The high-elevation satellite has its own advantages which can affect the success rate. Based on the E-ADOP, we can determine which factor is dominant in control of E-ADOP and ADOP in different conditions and to make ADOP different among BDS, GPS and BDS/GPS. The main conclusions, which are listed below, were verified by experiments with 5 km, 10 km and 15 km baselines in both clear sky conditions at Hong Kong Base Station and complex sky conditions in Xuzhou for BDS, GPS and BDS/GPS systems.

The main conclusions are as follows:

1. When new satellites are added, the ADOP becomes smaller mainly because the number of satellites becomes larger. On the other hand, when the number of satellites is constant, the greater the proportion of high-elevation satellites is, the smaller the ADOP is. Namely, ADOP is mainly affected by the SMRW.
2. In contrast to systems where the satellites with low-elevation are the majority or where low- and high-elevation satellites are equally distributed, in systems where the high-elevation satellites are the majority, the SMRW makes ADOP smaller even if there are fewer satellites than in the previous two cases; however, the difference in number of satellites should not be large and it can be further expanded as the proportion of high-elevation satellites becomes larger or the elevations of those satellites become larger.
3. The ADOP of BDS is smaller than ADOP of GPS mainly because of the SMRW or the large number of high-elevation satellites in BDS. The large number of satellites makes ADOP of BDS/GPS smaller and success rate of BDS/GPS higher than those of a single system.
4. According to the experimental results obtained in clear sky conditions, for 5 km, 10 km and 15 km baselines, the success rates of BDS/GPS are all larger than 99.00%, but the success rates of GPS are all less than 92.00%. For 5 km and 10 km baselines, success rates of BDS and BDS-E are all larger than 99.00% due to the large number of high-elevation satellites, while for 15 km baseline, their success rates are less than 98.00%. Hence, the baseline length of BDS that can be calculated (which means its success rate is larger than 99.00%) is about 10 km, the baseline length of GPS is shorter than 5 km, and the baseline length of BDS/GPS is longer than 15 km. Further, in complex sky conditions, the success rate and baseline accuracy of BDS are all superior to those of GPS due to numerous high-elevation satellites and therefore in complex sky conditions the ambiguity resolution performance of BDS is far better than that of GPS.

The BDS has a large number of high-elevation satellites because its unique constellation design includes GEO and IGSO satellites that are all high-orbit satellites, which can improve the success rate of SFSE ambiguity resolution for short baselines. We believe that the baseline length, which can be calculated using SFSE, can be further expanded. At the same time, this study has a guiding significance and provides the theoretical basis for both selection of satellites for a multi-system and improvement of success rate in complex sky conditions.

## Figures and Tables

**Table 1:** Zenith-referenced code and phase standard deviation.

System	Band	Code (cm)	Phase (mm)
GPS	L2	27	2.5
BDS	B2	30	2.5

**Table 2:** Number of satellites for different cut-off elevations.

Baseline (km)	System	Cut-Off Elevation							
		10	20	25	30	35	40	50	60
5	BDS	10.61	10.22	8.9	8.61	7.36	7.1	4.93	2.65
	GPS	9.01	7.12	6.23	5.35	4.48	3.75	2.41	1.17
	BDS/GPS	19.62	17.34	15.12	13.96	11.84	10.85	7.33	3.82
10	BDS	10.92	10.21	8.88	8.6	7.35	7.09	4.94	2.64
	GPS	9.02	7.11	6.22	5.35	4.48	3.75	2.42	1.17
	BDS/GPS	19.95	17.33	15.11	13.96	11.84	10.85	7.35	3.81
15	BDS	10.92	10.21	8.88	8.6	7.35	7.09	4.9	2.65
	GPS	9.02	7.11	6.22	5.35	4.48	3.75	2.41	1.17
	BDS/GPS	19.95	17.33	15.11	13.96	11.84	10.85	7.31	3.81

**Table 3:** Results of BDS, BDS-E, GPS and BDS/GPS for 5 km baseline.

	System	High-Elevation/Total Satellite Number	$SMRW^{\frac{1}{2}}$/β	ADOP/E-ADOP	$P_{s,IB}$ (%)	$P_{s,E}$ (%)	PDOP	Accuracy (cm)
A	BDS	7.3/10.7	446/5.4	0.112/2.04	99.84	99.99	2.03	1.11
	BDS-E	6.9/8.9	65/3.7	0.148/2.29	96.28	99.95	3.14	1.61
	GPS	4.1/8.9	2517/6.7	0.206/2.63	83.00	87.12	1.93	1.50
	BDS/GPS	11.4/19.6	501,926/11.7	0.070/1.47	100.00	100.00	1.23	0.90
B	BDS	7.1/10.2	289/4.9	0.119/2.06	99.68	100.00	2.2	1.37
	BDS-E	6.3/8.9	98/4.0	0.146/2.32	98.20	99.95	2.54	1.51
	GPS	4.3/8.9	2530/6.64	0.206/2.62	83.05	91.27	1.93	1.36
	BDS/GPS	11.4/19.1	224,579/11.2	0.071/1.48	100.00	100.00	1.25	1.15

**Table 4:** Results of BDS, BDS-E, GPS and BDS/GPS for 10 km baseline.

	System	High-Elevation/Total Satellite Number	$SMRW^{\frac{1}{2}}$/β	ADOP/E-ADOP	P_s,IB_ (%)	P_s,E_ (%)	PDOP	Accuracy (cm)
A	BDS	7.3/10.8	522/5.6	0.112/2.04	99.88	99.99	2.01	1.65
	BDS-E	6.9/8.9	65/3.7	0.148/2.29	96.24	99.88	3.14	2.13
	GPS	4.1/8.9	2609/6.7	0.206/2.62	82.97	83.51	1.93	1.82
	BDS/GPS	11.4/19.7	509597/11.9	0.070/1.47	100.00	100.00	1.22	1.39
B	BDS	7.9/10.9	425/5.3	0.109/2.01	99.89	100.00	2.35	2.49
	BDS-E	7.0/8.7	43/3.4	0.150/2.3	95.20	99.19	3.40	2.79
	GPS	4.3/8.8	2667/6.5	0.212/2.65	81.44	87.24	1.98	2.20
	BDS/GPS	12.2/19.7	395,365/11.5	0.069/1.45	100.00	100.00	1.28	1.92

**Table 5:** Results of BDS, BDS-E, GPS and BDS/GPS for 15 km baseline.

	System	High-Elevation/Total Satellite Number	$SMRW^{\frac{1}{2}}$/β	ADOP/E-ADOP	*P*_s,IB_ (%)	$P_{s,E}$ (%)	PDOP	Accuracy (cm)
A	BDS	7.2/9.5	162/4.4	0.133/2.21	98.00	97.12	2.21	2.95
	BDS-E	7.2/8.9	75/3.6	0.141/2.27	97.21	96.13	2.51	3.34
	GPS	4.3/8.9	2482/6.5	0.209/2.63	82.00	76.61	1.94	2.85
	BDS/GPS	11.5/18.4	250,572/10.6	0.073/1.51	100.00	100.00	1.32	2.19
B	BDS	7.2/10.0	372/4.9	0.127/2.17	98.79	95.33	2.64	3.40
	BDS-E	7.2/8.9	59/3.6	0.140/2.27	97.98	89.25	3.52	3.62
	GPS	4.3/8.9	3080/6.56	0.212/2.65	81.42	62.48	1.96	2.73
	BDS/GPS	11.5/18.9	384,463/11.1	0.073/1.51	100.00	100.00	1.31	2.60

**Table 6:** Results of BDS, BDS-E, GPS and BDS/GPS.

System	High-Elevation/Total Satellite Number	$SMRW^{\frac{1}{2}}$/β	ADOP/E-ADOP	*P*_s,IB_ (%)	$P_{s,E}$ (%)	PDOP	Accuracy (cm)
BDS	8.0/10.0	57/4.0	0.111/2.05	99.81	99.83	2.76	1.22
BDS-E	8.0/8.5	21/3.0	0.141/2.30	99.23	99.12	3.40	1.22
GPS	4.6/8.5	1023/6.0	0.217/2.73	82.04	86.45	1.91	1.69
BDS/GPS	12.6/18.5	41,424/9.6	0.069/1.45	99.95	100.00	1.35	1.33

**Table 7:** Results of BDS, BDS-E, GPS and BDS/GPS.

System	High-Elevation/Total Satellite Number	$SMRW^{\frac{1}{2}}$/*β*	ADOP/E-ADOP	$P_{s,IB}$ (%)	$P_{s,E}$ (%)	PDOP	Accuracy (cm)
BDS	5.9/9.9	1479/6.4	0.148/2.34	98.59	99.68	2.48	1.01
BDS-E	5.9/8.8	161/5.0	0.168/2.49	95.5	98.99	2.66	1.01
GPS	4.0/8.8	3144/6.74	0.212/2.67	80.66	92.76	1.9	1.72
BDS/GPS	9.9/18.7	3,434,713/13.0	0.082/1.62	99.94	100	1.28	1.48

## Appendix A

**Proof** **of** **Theorem** **1.** Based on the condition $0<w_{s}\leq 1$, it is easy to get:

(A1) $$\sum_{s=1}^{m}w_{s}+\sum_{s=m+1}^{z}w_{s}>\sum_{s=1}^{m}w_{s}$$

(A2) $$\prod_{s=1}^{m}w_{s}\cdot \prod_{s=m+1}^{z}w_{s}\leq \prod_{s=1}^{m}w_{s}$$

where $m\in N^{*}$, $z\in N^{*}$ and m<z. Combining Equations (A1) and (A2), Equation (A3) can be written:

(A3) $$A=\frac{\sum_{s=1}^{m}w_{s}+\sum_{s=m+1}^{z}w_{s}}{\prod_{s=1}^{m}w_{s}\cdot \prod_{s=m+1}^{z}w_{s}}>B=\frac{\sum_{s=1}^{m}w_{s}}{\prod_{s=1}^{m}w_{s}}$$

For $0<w_{s}\leq 1$, Equation (A4) can be gotten:

(A4) $$\frac{A}{B}=\frac{\sum_{s=1}^{m}w_{s}+\sum_{s=m+1}^{z}w_{s}}{\prod_{s=1}^{m}w_{s}\cdot \prod_{s=m+1}^{z}w_{s}}\cdot \frac{\prod_{s=1}^{m}w_{s}}{\sum_{s=1}^{m}w_{s}}=\frac{1}{\prod_{s=m+1}^{z}w_{s}}+\frac{\sum_{s=m+1}^{z}w_{s}}{\left(\prod_{s=m+1}^{z}w_{s}\right)\cdot \left(\sum_{s=1}^{m}w_{s}\right)}>\frac{1}{\prod_{s=m+1}^{z}w_{s}}$$

According to Equations (A3) and (A4), the SMRW becomes larger when some $w_{s}$ are added, and the magnification is greater by $\frac{1}{\prod_{m+1}^{z}w_{s}}$ times. End of Proof.

**Proof** **of** **Theorem** **2.** This theorem is proven by mathematical induction. Let $B=\frac{\sum_{s=1}^{m}w_{s}}{\prod_{s=1}^{m}w_{s}}$ and $C=\frac{\sum_{i=1}^{m}w_{i}}{\prod_{i=1}^{m}w_{i}}$. Firstly, when m=1, it is easy to get:

(A5) B=C=1

Hence, when m=1, Theorem 2 holds. Secondly, suppose that when m=h and h>1, Theorem 2 also holds:

(A6) $$\frac{\sum_{s=1}^{h}w_{s}}{\prod_{s=1}^{h}w_{s}}\leq \frac{\sum_{i=1}^{h}w_{i}}{\prod_{i=1}^{h}w_{i}}$$

Lastly, when m=h+1, B and C can be written as:

(A7) $$B=\frac{\left(\sum_{s=1}^{h}w_{s}\right)+w_{s=h+1}}{\left(\prod_{s=1}^{h}w_{s}\right)\cdot w_{s=h+1}}$$

(A8) $$C=\frac{\left(\sum_{i=1}^{h}w_{i}\right)+w_{i=h+1}}{\left(\prod_{i=1}^{h}w_{i}\right)\cdot w_{i=h+1}}$$

Based on the Equation (A4), compared to B and C when *m* is equal to *h*, the B has expanded $\frac{1}{w_{s=h+1}}+\frac{1}{\sum_{s=1}^{h}w_{s}}$ times and C has expanded $\frac{1}{w_{i=h+1}}+\frac{1}{\sum_{i=1}^{h}w_{i}}$ times when *m* is equal to *h* + 1. For $\forall s\in N^{*}$ and $\forall i\in N^{*}$, when s=i, $w_{s}$ is always greater than or equal to $w_{i}$ after $w_{s}$ and $w_{i}$ are sorted from small to large, respectively. Hence, Equations (A9) and (A10) can be easily obtained:

(A9) $$w_{s=h+1}\geq w_{i=h+1}$$

(A10) $$\sum_{s=1}^{h}w_{s}\geq \sum_{i=1}^{h}w_{i}$$

Equations (A9) and (A10) can be rewritten:

(A11) $$\frac{1}{w_{s=h+1}}\leq \frac{1}{w_{i=h+1}}$$

(A12) $$\frac{1}{\sum_{s=1}^{h}w_{s}}\leq \frac{1}{\sum_{i=1}^{h}w_{i}}$$

Based on Equations (A11) and (A12), Equation (A13) can be obtained:

(A13) $$\frac{1}{w_{s=h+1}}+\frac{1}{\sum_{s=1}^{h}w_{s}}\leq \frac{1}{w_{i=h+1}}+\frac{1}{\sum_{i=1}^{h}w_{i}}$$

According to Equation (A13), the expanded times of C is larger than that of B. Combined with Equation (A6), we can get:

(A14) $$\frac{\left(\sum_{s=1}^{h}w_{s}\right)+w_{s=h+1}}{\left(\prod_{s=1}^{h}w_{s}\right)\cdot w_{s=h+1}}\leq \frac{\left(\sum_{i=1}^{h}w_{i}\right)+w_{i=h+1}}{\left(\prod_{i=1}^{h}w_{i}\right)\cdot w_{i=h+1}}$$

Therefore, when *s* and *i* are equal to *h* + 1, Theorem 2 holds. Hence, for $\frac{\sum_{s=1}^{m}w_{s}}{\prod_{s=1}^{m}w_{s}}$ and $\frac{\sum_{i=1}^{m}w_{i}}{\prod_{i=1}^{m}w_{i}}$, if $w_{s}\geq w_{i}$, when *s* = *i* (*i* = 1, 2, …, *m*) after $w_{s}$ and $w_{i}$ are sorted from small to large, respectively, $\frac{\sum_{s=1}^{m}w_{s}}{\prod_{s=1}^{m}w_{s}}$ is less than or equal to $\frac{\sum_{i=1}^{m}w_{i}}{\prod_{i=1}^{m}w_{i}}$. End of Proof.

**Proof of an Important Property for E-ADOP**  According to Equation (11), the mean ADOP can be written as:

(A15) $$\bar{\mathrm{ADOP}}=\frac{\sum_{i=1}^{M}f_{1}^{i}\cdot f_{2}^{i}\cdot f_{3}^{i}}{M}$$

where *M* is the number of epochs. $f_{2}$ and $f_{3}$ are the form of power exponential function of number of satellites and SMRW and the form of exponential function of number of satellites. Hence, in general:

(A16) $$\bar{\mathrm{ADOP}}=\frac{\sum_{i=1}^{M}f_{1}^{i}\cdot f_{2}^{i}\cdot f_{3}^{i}}{M}\neq \frac{\sum_{i=1}^{M}f_{1}^{i}}{M}\cdot \frac{\sum_{i=1}^{M}f_{2}^{i}}{M}\cdot \frac{\sum_{i=1}^{M}f_{3}^{i}}{M}$$

For E-ADOP, according to Equation (16), the mean E-ADOP can be written as:

(A17) $$\bar{E–\mathrm{ADOP}}=\frac{\sum_{i=1}^{M}\alpha_{i}\cdot \left(\beta_{i}+\xi \right)}{M}=\frac{\sum_{i=1}^{M}\alpha_{i}\cdot \beta_{i}}{M}+\frac{\xi \cdot \sum_{i=1}^{M}\alpha_{i}}{M}$$

Most of the time, the number of satellites *m* in GPS are between 8 and 9, which means $\alpha =\frac{1}{m-1}$ is between 0.125 and 0.143. When the mean value (8.5) of number of satellites is used, $\alpha^{\prime }=\frac{1}{m-1}$ is about 0.133. When α and $\alpha^{\prime }$ are brought into Equation (A17) to obtain $\bar{E–\mathrm{ADOP}}$ and ${\bar{E–\mathrm{ADOP}}}^{\prime }$, and the difference between $\bar{E–\mathrm{ADOP}}$ and ${\bar{E–\mathrm{ADOP}}}^{\prime }$ is small due to the small difference between α and $\alpha^{\prime }$, which also applies to BDS. Hence, the $\alpha_{i}$ (*i* = 1, 2, …, *M*) in Equation (A17) can be replaced by a constant *α,* which is calculated by mean number of satellites. Then, Equation (A17) can be rewritten:

(A18) $$\bar{E–\mathrm{ADOP}}=\frac{\sum_{i=1}^{M}\alpha_{i}\cdot \beta_{i}}{M}+\frac{\xi \cdot \sum_{i=1}^{M}\alpha_{i}}{M}\approx \frac{\alpha \cdot \sum_{i=1}^{M}\beta_{i}}{M}+\alpha \cdot \xi =\alpha \cdot \left(\bar{\beta }+\xi \right)$$

Hence, the mean E-ADOP value can be predicted more accurately by the mean value of parameters through the expression of E-ADOP than through the ADOP expression for mean ADOP value. End of Proof.
